# Fungal and bacterial pathogenic co-infections mainly lead to the assembly of microbial community in tobacco stems

**DOI:** 10.1515/biol-2025-1103

**Published:** 2025-09-20

**Authors:** Can Wang, Zhipeng Xiao, Zhihui Cao, Feng Sheng, Penghua Xiang, Tingting Mu, Yunming Ma, Xuliang Lin, Mengyu Xiao, Qian Zhu, Shaolong Wu, Lin Tan

**Affiliations:** Hengyang Tobacco Company of Hunan, Hengyang, Hunan, China; Tobacco Company of Hunan, Changsha, Hunan, China; College of Plant Protection, Hunan Agricultural University, Changsha, Hunan Province, China

**Keywords:** co-infection, microbiomes, pathogen interactions, tobacco

## Abstract

Pathogenic co-infections in plants significantly impact microbial diversity and disease outcomes, yet their effects on microbial community structure and ecological processes remain unclear. Tobacco plants were co-infected with *Ralstonia solanacearum* and *Neocosmospora falciformis*. 16S ribosomal RNA and internal transcribed spacer amplicon sequencing were used to assess bacterial and fungal communities, respectively, in infected tobacco stems. The results were compared between co-infected and healthy control tobacco plants to assess the effects of infection. Co-infection reduced microbial diversity and shifted community structure, promoting ecological specialization. Network analysis revealed synergistic interactions between the pathogens, enhancing virulence through positive correlations with certain microbial taxa. Conversely, some taxa exhibited antagonistic effects, potentially limiting pathogen proliferation. Deterministic processes were found to dominate microbial community assembly under infection conditions, significantly reshaping the microbial landscape compared to healthy control plants. This study highlights the profound effects of co-infection on microbial diversity, community composition, microbial interactions, and community assembly processes in tobacco plants. These findings provide valuable insights for developing more targeted plant disease management strategies by manipulating microbial communities.

## Graphical abstract

**Figure:**
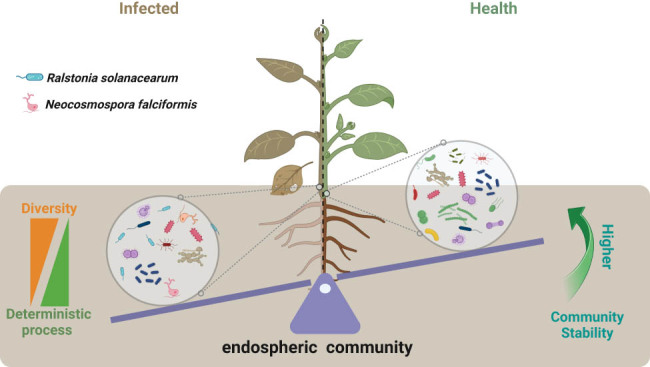

## 1 Introduction

Tobacco (*Nicotiana tabacum*) is a vital cash crop on a global scale, delivering substantial economic benefits and sustaining the livelihoods of millions of farmers worldwide [1]. Its cultivation and processing are integral to the agricultural economies of numerous countries, where it contributes significantly not only through direct income generation for growers but also by supporting ancillary industries such as manufacturing, processing, and trade [2]. The economic impact of tobacco extends beyond primary agriculture, influencing market dynamics and employment across various sectors, including transportation, retail, and export services [3,4]. The widespread cultivation and trade of tobacco products also generate significant tax revenues, which are crucial for the economies of many developing and developed nations [5]. However, the cultivation of tobacco faces persistent challenges, particularly from plant diseases that threaten crop yield and quality [6,7]. These diseases can cause severe economic losses, not only by reducing the marketable output but also by necessitating increased costs for disease management and control measures [8].

In recent years, the prevalence and severity of tobacco diseases have been exacerbated by several contributing factors, including agricultural intensification, monoculture practices, and climate change [9]. The intensification of agriculture, characterized by increased use of inputs such as fertilizers and irrigation, can create more favorable conditions for pathogen proliferation [10]. Monoculture practices, where large areas are planted with the same crop variety, can lead to the buildup of specific pathogens in the soil and increase the risk of disease outbreaks [11]. Additionally, climate change, with its associated shifts in temperature, humidity, and precipitation patterns, can alter the epidemiology of plant diseases, influencing the incidence, distribution, and severity of infections [12,13]. These factors collectively create a more conducive environment for the emergence and spread of complex disease scenarios, including co-infection. Co-infection, defined as the simultaneous infection of a host by multiple pathogens [14,15], presents a particularly challenging aspect of disease management in tobacco cultivation. This phenomenon is of concern because it can lead to increased disease severity and complicate management strategies. Co-infections can result in complex interactions between pathogens, which may synergistically exacerbate the damage to the plant, increase the virulence of the disease, or alter the effectiveness of control measures [16,17]. Understanding the dynamics of co-infection is crucial for developing effective strategies to mitigate the impact of these diseases on tobacco production.

Among the various pathogens that can infect tobacco, the bacterial pathogen *Ralstonia solanacearum* [18] and the fungal pathogens *Neocosmospora falciformis* [19] are particularly notorious for their pathogenicity and impact. *R. solanacearum* is a soil-borne bacterium that causes bacterial wilt, a devastating disease characterized by the wilting and eventual death of the plant [6,20]. This pathogen is highly virulent and poses a significant threat to tobacco crops due to its ability to persist in soil and water environments. It invades the plant’s vascular system, disrupting water and nutrient transport, which leads to systemic infection and severe wilting symptoms [21]. The control of *R. solanacearum* is challenging due to its broad host range and the difficulty of eradicating it from contaminated soil. *N. falciformis*, another significant pathogen, is known for causing root rot [22]. This disease affects the plant’s endospheric system, impairing its ability to uptake water and nutrients, which results in stunted growth and reduced vigor [19]. The persistence of *N. falciformis*, formerly classified under the genus *Fusarium*, in soil and plant debris presents a continuous challenge for long-term management in infested fields [19]. This pathogen is known to frequently cause root rot diseases in Solanaceae, significantly impacting crop health [23]. Its presence can lead to substantial reductions in both the aesthetic and market value of affected crops due to the visible damage it inflicts on foliage and stems. Furthermore, when *N. falciformis* co-infects tobacco plants along with other pathogens, the resulting disease complex can be more severe than the sum of individual infections [16]. Co-infection can enhance pathogenicity through synergistic interactions, where the presence of one pathogen may facilitate the colonization or increase the virulence of another [24,25]. Such interactions can complicate disease management, as they may lead to unexpected disease outcomes and necessitate different management strategies compared to those used for single pathogen infections. Despite the clear implications of co-infection for disease severity and management, there is a significant gap in our understanding of how these pathogens interact within the tobacco host [26]. The specific interactions between *R. solanacearum* and *N. falciformis* during co-infection are not well-characterized. Previous studies have suggested that co-infection can lead to altered disease progression and severity, potentially resulting in increased virulence, changes in symptom expression, and enhanced transmission potential of the pathogens [27,28]. However, the underlying mechanisms driving these interactions remain poorly understood [15]. This study aims to fill this gap by investigating the dynamics of *R. solanacearum* and *N. falciformis* co-infection in tobacco plants. The hypotheses are (1) co-infection significantly alters microbial community structure and function in tobacco stems, (2) synergistic interactions between these pathogens exacerbate disease severity, and (3) microbial community assembly processes, shaped by deterministic ecological factors, influence the outcome of co-infection.

Network analysis provides a robust framework for modeling and understanding the complex relationships between different organisms within a shared environment [29]. By constructing interaction networks, we can identify key nodes – such as specific pathogens or host responses – that play critical roles in the disease process [6]. This approach not only helps in understanding the underlying mechanisms of co-infection but also in identifying potential targets for intervention. For example, network analysis can reveal keystone pathogens or microbial communities that disproportionately influence the overall health of the plant [30]. Moreover, network analysis can uncover hidden patterns of interaction that are not apparent through traditional analytical methods [31]. For instance, it can help identify indirect interactions where one pathogen’s presence modifies the host environment in a way that benefits or hinders another pathogen [32]. These insights are crucial for developing targeted management strategies, such as the introduction of beneficial microorganisms that can suppress pathogen activity or enhance plant resilience [33,34]. Ecologically, network analysis helps to understand how microbial community assembly processes are influenced under co-infection scenarios, where multiple pathogens interact with one another and with the host environment. These interactions can drive shifts in community composition, leading to either beneficial or detrimental ecological outcomes for the plant. Understanding these assembly processes can reveal the balance between microbial cooperation and competition, which is crucial for the resilience of plant microbiomes [35]. In agricultural settings, this knowledge enables the identification of microbial community configurations that favor disease resistance or enhance plant health, offering a foundation for strategies that can manipulate microbial diversity to achieve more sustainable disease control and improve crop productivity [36].

The primary objectives of this study are (1) to investigate the microbial interactions in tobacco stems with *R. solanacearum* and *N. falciformis* co-infection, (2) to elucidate the mechanisms through which co-infection influences disease severity and progression, and (3) to identify beneficial microorganisms that can potentially mitigate the effects of these pathogens. Understanding these pathogen interactions is essential for improving diagnostic accuracy and refining treatment strategies. The outcomes of this study are expected to provide valuable insights into the complex dynamics of co-infection, contributing to the development of integrated pest management strategies that are more effective and sustainable. Such strategies could enhance the resilience of tobacco cultivation, safeguard yields, and improve the economic viability of the crop in the face of emerging disease challenges.

## 2 Materials and methods

### 2.1 Study site and soil sampling

The study was conducted in the tobacco field of Ji Ling village, Ma Shui Town, Leiyang city, Hunan Province, China. This region experiences a subtropical monsoon humid climate with an average annual temperature of 17.9°C, 1,640 h of sunshine, and an average annual rainfall of 1,337 mm. The soil type is predominantly loam, according to the FAO classification [37]. Fieldwork was performed in late July 2022. Since 2003, the fields in this area have followed a tobacco–rice rotation cropping system. The experimental field covered an area of over 667 m^2^ and was managed using uniform agricultural practices. For pathogen inoculation, tobacco plants were exposed to *R. solanacearum* and *N. falciformis*. Both pathogens were inoculated via root dipping. A bacterial suspension of *R. solanacearum* was prepared at a concentration of 10^8^ CFU/mL and applied to the tobacco plants’ root systems. *N. falciformis* was inoculated by introducing a spore suspension at a concentration of 10^6^ spores/mL into the root zone. Inoculations were performed simultaneously to ensure uniform exposure to both pathogens. For endospheric sample preparation, tobacco plants’ stems exhibiting symptoms of co-infection by *R. solanacearum* and *N. falciformis* were selected based on disease (IR) signs observed in the field. An equal number of healthy tobacco plants stems (HR), showing no signs of infection, were also collected for comparison. All tobacco stems were washed sequentially with 75% ethanol, 2.5% sodium hypochlorite, and sterile water [6]. The stems were then cut into small pieces, homogenized in a mortar with PBS, and the mixture was transferred to centrifuge tubes. After standing for 30 min, the homogenate was centrifuged to remove the supernatant [7]. The resulting cell pellets (endophytic samples) were stored at −80°C until DNA extraction.

### 2.2 DNA extraction and amplicon sequencing

A total of 16 endospheric samples were processed consisting of both healthy and infected tobacco stems, with eight samples from each group. DNA extraction was performed using the FastDNA^TM^ SPIN Kit (MP Biomedicals) following the manufacturer’s protocol. The quantity and quality of the extracted DNA were evaluated using a NanoDrop Spectrophotometer (Nano-100, Aosheng Instrument Co. Ltd), ensuring that the A260/A280 ratios fell between 1.8 and 2.0, indicating good-quality DNA suitable for downstream applications. For bacterial amplicon sequencing, the V5–V6 region of the 16S ribosomal RNA (rRNA) gene was targeted to avoid amplification of chloroplast DNA. Universal primers 799F (5′-AACMGGATTAGATACCCKG-3′) and 1115R (5′-AGGGTTGCGCTCGTTG-3′) were used for amplification [6]. For fungal amplicon sequencing, the internal transcribed spacer (ITS) regions were amplified using the primers 5.8F (5′-AACTTTYRRCAAYGGATCWCT-3′) and 4R (5′-AGCCTCCGCTTATTGATATGCTTAART-3′) [38]. To facilitate high-throughput sequencing, 12 bp unique barcodes were added to the 5′-ends of both forward and reverse primers for sample identification. The PCR reactions were performed in a 50 µL reaction mixture containing 1.5 µL of dNTP mixture, 0.5 µL Taq DNA polymerase (TaKaRa, Beijing, China), 5 µL of 10× PCR buffer, 1.5 µL of each 10 µM primer, and 20–30 ng of DNA template. The thermal cycling conditions were as follows: initial denaturation at 94°C for 1 min, followed by 30 cycles of 94°C for 20 s, 57°C for 25 s, and 68°C for 45 s. A final extension at 72°C for 10 min was followed by a hold at 4°C. The amplification products were verified via gel electrophoresis to ensure successful amplification. Following amplification, the PCR products were purified using the Agencourt AMPure XP beads (Beckman Coulter, USA) to remove primer dimers and non-specific amplification products. The purified amplicons were then quantified using the Qubit 3.0 fluorometer (Thermo Fisher Scientific, USA) with the Qubit dsDNA HS Assay Kit. Equal amounts of purified amplicons from each sample were pooled, and the pool was subjected to quality control using the Agilent 2100 Bioanalyzer (Agilent Technologies, USA) to confirm the correct amplicon size distribution. Sequencing was performed using the Illumina HiSeq 2500 platform (Illumina, USA) with paired-end 250-bp reads. This high-throughput sequencing method ensured the comprehensive profiling of the microbial communities. All sequencing was conducted by Magigene Biotechnology Co., Ltd (Guangzhou, China), with the sequencing data processed and analyzed using standard bioinformatics pipelines.

### 2.3 Sequence processing

A total of 32 samples, including 16 bacterial community samples and 16 fungal community samples, were analyzed. All raw reads from the 16S rRNA and ITS regions were uploaded to a comprehensive sequence analysis pipeline, which integrates multiple bioinformatics tools [31]. This pipeline, hosted on a private network, facilitates internal data processing and analysis. Initially, the raw reads were assigned to samples based on their barcodes using the “Detected barcodes” tool, followed by trimming of the barcode sequences [39]. Subsequently, the FLASH tool was employed to merge forward and reverse reads. The merged reads, devoid of ambiguous bases, were filtered using the Btrim tool [40]. For chimera checking, the Greengenes database was used for bacterial communities [41], and the ITS RefSeq database was used for fungal communities [42]. Singletons were retained to preserve rare species, and sequences were clustered into zero-radius operational taxonomic units (zOTUs) at a 97% similarity threshold using Unoise. For ITS gene sequences, the ITSx tool was utilized to identify and extract the ITS regions. Following the generation of OTU tables, reads were randomly resampled to 100,000 sequences per bacterial sample and 25,000 sequences per fungal sample. The Ribosomal Database Project classifier was used to assign bacterial and fungal OTUs with the Greengenes ribosomal database and UNITE database, respectively, ensuring confidence values greater than 0.8.

### 2.4 Network analysis using random matrix theory (RMT) and evaluation of community stability

RMT was utilized to construct bacterial community networks based on Spearman correlations, leveraging an open-access analysis pipeline available at https://inap.denglab.org.cn/ [43]. Spearman correlation coefficients were filtered using thresholds of *r* > 0.91 for bacterial communities and *r* > 0.85 for fungal communities, with a false discovery rate of <0.05. These stringent thresholds were chosen to ensure the robustness and reliability of the network connections. Applying consistent thresholds across all treatments allowed for direct comparisons of network properties, ensuring that any observed differences were attributable to treatment effects rather than variations in network construction parameters. The resulting networks were visualized using Gephi (version 0.9.2). To characterize the topological structure of the microbial ecological networks (MENs), various indices were measured, including the number of nodes and links, power-law fitting of node degrees, average degree (avgK), average clustering coefficient (avgCC), and modularity. Synergistic interactions were defined as positive correlations (*r* > 0.91 for bacteria and *r* > 0.85 for fungi), where the presence of one microorganism enhanced the growth or activity of another, suggesting a cooperative relationship. Antagonistic interactions were defined as negative correlations (*r* < −0.91 for bacteria and *r* < −0.85 for fungi), where the presence of one microorganism inhibited the growth or activity of another, indicating competition or inhibition. Statistical significance of these interactions was assessed by comparing the observed correlation coefficients to the null distribution generated by randomly rewiring the network 100 times using the Maslov–Sneppen approach [44]. A correlation coefficient was considered statistically significant if it exceeded the 95th percentile of the null distribution. For each observed MEN, the Maslov–Sneppen approach was employed to generate 100 randomly rewired networks to compare against the observed network properties.

Robustness indices were used to assess the resistance of the microbial community. The evaluation process involved two key steps: (i) calculation of abundance-weighted mean interaction strength (wMIS_*i*_). The wMIS_*i*_ for node *i* was calculated using the following equation:

(1) \[{{\mathrm{wMIS}}}_{i}=\frac{\sum _{j\ne i}{b}_{j}{s}_{{ij}}}{{\sum }_{j\ne i}{b}_{j}},]\]

where *b*_*j*_ is the relative abundance of species *j* and *s*_*ij*_ is the association strength between species *i* and *j*, measured by Pearson correlation coefficient. (ii) Network robustness assessment. Nodes with wMIS_*i*_ values of 0 were removed from the network. The fraction of the remaining nodes after this elimination process was reported as the network robustness. By following these steps, the robustness of the microbial network was quantitatively evaluated, providing insights into the community’s resistance to disturbances and its overall stability.

### 2.5 Statistical analysis

All statistical analyses were performed using the analysis pipeline available at https://dmap.denglab.org.cn/. Alpha diversity, representing within-sample diversity, was evaluated using indices such as Shannon, Simpson, and Chao1. Beta diversity, which measures between-sample diversity, was analyzed using Bray–Curtis dissimilarity and UniFrac distances. To assess community dissimilarity, we employed multiple methods: multi-response permutation procedure, analysis of similarities, and permutational multivariate analysis of variance. Prior to conducting these analyses, we confirmed that soil physicochemical variables and diversity indices followed a normal distribution. These analyses provide insights into the diversity and compositional differences within and between microbial communities. To pinpoint specific differences between group means, we utilized both the least significant difference test and Tukey’s *post-hoc* test. The structural features of the observed networks were compared to those of random networks using a one-sample Student’s *t*-test, ensuring a robust statistical framework for interpreting network differences.

Moreover, to quantify the niche width of microbial communities, we utilized Levins’ niche breadth (*B*) index, as described by Logares et al. [45] and Jiao et al. [46]. The index is calculated using the following formula:

(2) \[B=\frac{1}{\sum _{i}{p}_{i}^{2}},]\]

where *p*_*i*_ is the proportion of individuals using resource *i*. This method allows for the assessment of the range of conditions and resources that microbial species can utilize, providing insights into their ecological versatility and adaptability.

## 3 Results

### 3.1 Substantial shifts in pathogenic bacteria and fungi under infected conditions

**Figure 1:**
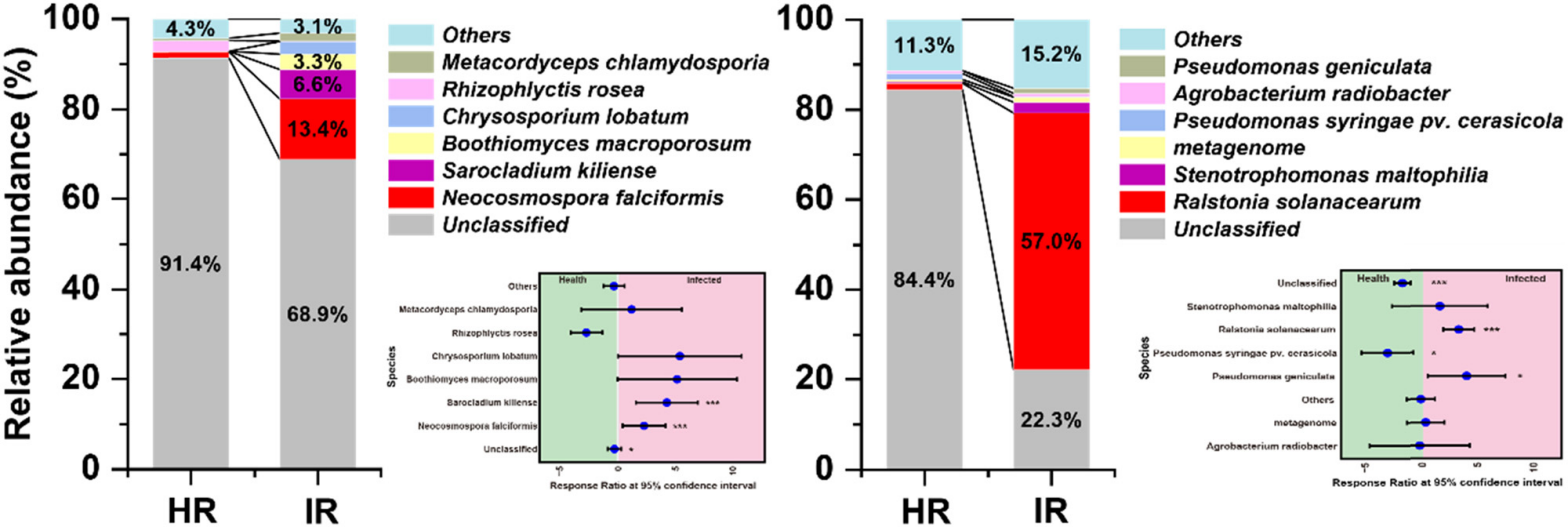
Changes in pathogenic microbiome composition. The left panel illustrates the composition changes and response ratios of the fungal pathogens *N. falciformis* and *Sarocladium kiliense*. The right panel shows the composition changes and response ratios of the bacterial pathogen *R. solanacearum*. Statistical significance is indicated as follows: *P* < 0.05 (*), *P* < 0.01 (**), and *P* < 0.001 (***).

In the comparison of microbial community composition between healthy (HR) and infected stems (IR), significant shifts in the relative abundance of key pathogenic bacteria were observed. *R. solanacearum*, a primary bacterial pathogen, exhibited a dramatic increase in abundance (*P* < 0.001), constituting 56.97% of the bacterial community in infected stems, compared to a minimal presence in healthy stems (1.44%). This substantial rise underscores *R. solanacearum*’s dominant role in the microbial community under disease conditions. Similarly, *N. falciformis* another fungal pathogen associated with plant diseases, showed a marked increase in infected stems (*P* < 0.001), rising from 1.33% in healthy samples to 13.36% in infected samples (Figure 1). This suggests that *R. solanacearum* and *N. falciformis* gains a competitive advantage or thrives under the altered environmental conditions present in infected stems.

### 3.2 Impact of infection on microbial diversity and community structure in plant stems

Using a 97% similarity threshold, our analysis identified 10,044 bacterial zOTUs and 1,115 fungal zOTUs. Notably, the Chao1 index and observed richness for fungal communities were significantly higher in healthy stems (Chao1: 262, richness: 222) compared to infected stems (Chao1: 214, richness: 167), whereas no significant differences were observed between healthy (Chao1: 1,887, richness: 1,729) and infected (Chao1: 1,766, richness: 1,643) stems in the bacterial community (Figure S2, ANOVA, *P*  <  0.005). The Venn diagram analysis further illustrated the unique and shared OTUs within the bacterial and fungal communities of healthy and infected stems. In the bacterial community, healthy stems contained 4,090 unique OTUs, while infected stems had 3,159 unique OTUs, with 2,795 OTUs shared between both conditions (Figure S3(a)). Similarly, in the fungal community, healthy stems harbored 489 unique OTUs, and infected stems contained 400 unique OTUs, with 226 OTUs common to both (Figure S3(b)). These findings underscore the distinct microbial compositions associated with health and disease states, as well as the presence of microbial taxa that persist regardless of infection status.

Ordination analyses, visualized through non-metric multidimensional scaling (NMDS) based on Bray–Curtis dissimilarity and detrended correspondence analysis (DCA) plots, revealed clear differences in microbial community structures between HR and IR. In the NMDS plots (Figure S4(a) and (c)), the separation between microbial communities in HR (blue triangles) and IR (red circles) was pronounced, with distinct clustering patterns and low stress values (stress = 0.084 and 0.001), indicating a strong fit for the data. These clusters suggest that infection significantly alters microbial composition, leading to the emergence of distinct communities in infected samples compared to healthy ones. The DCA plots (Figure S4(b) and (d)) reinforced these observations, showing a marked divergence between HR and IR microbial communities along the primary ordination axes. The spread and separation of data points in these plots highlight the extent of compositional shifts driven by infection. Further analysis using dissimilarity tests revealed significant differences between healthy and infected soil (Tables S1 and S2). Overall, these results indicate that infection not only impacts microbial diversity but also induces substantial changes in community structure, resulting in distinctly different microbial ecosystems in infected stems compared to healthy ones.

### 3.3 Shifts in microbial community composition and structure between healthy and infected stems

**Figure 2:**
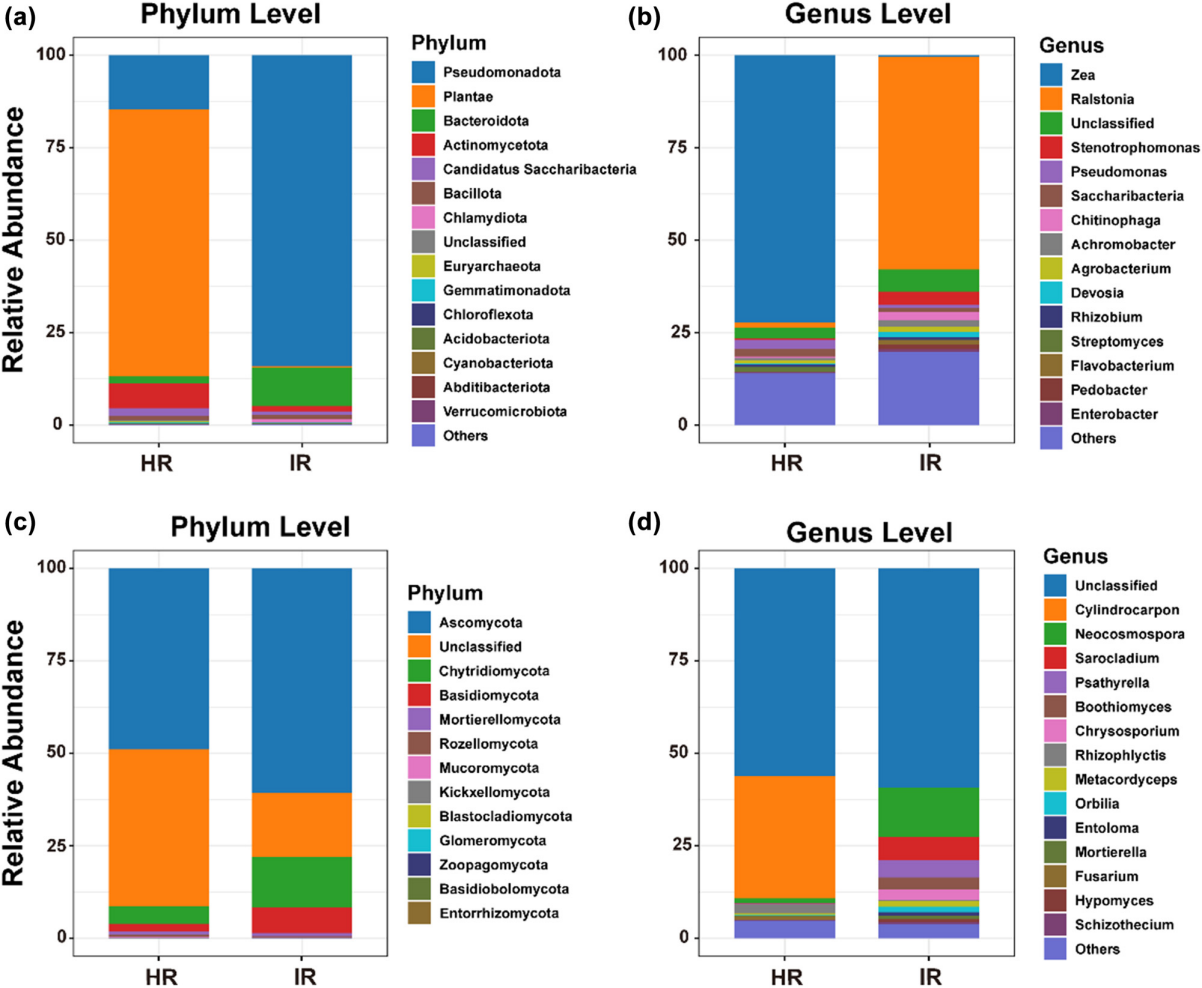
Composition changes in pathogenic microbiome at phylum and genus levels. (a) and (c) Relative abundance of bacterial and fungal communities at the phylum level in healthy stem (HR) and infected stem (IR). (b) and (d) Composition changes at the genus level for bacterial and fungal communities in HR and IR.

The comparison of microbial community composition between HR and IR at both phylum and genus levels reveals significant shifts in microbial taxa associated with infection. At the phylum level, the bacterial community in HR is predominantly composed of *Pseudomonadota*, followed by *Bacteroidota* and *Actinomycetota* (Figure 2(a) and (c)). However, in IR, there is a noticeable shift, with an increase in the relative abundance of *Pseudomonadota*, while other phyla such as *Bacteroidota* and *Actinomycetota* see a reduction. In the fungal community, *Ascomycota* remains dominant in both HR and IR, but shifts in the relative abundance of other phyla, such as *Mortierellomycota* and *Chytridiomycota*, are observed. At the genus level, *Ralstonia*, a known pathogen, shows a significant increase in IR, reflecting its critical role in disease development (Figure 2(b) and (d)). Other genera, such as *Stenotrophomonas* and *Pseudomonas*, also see an increase in infected samples, while the abundance of beneficial or neutral genera decreases. In the fungal community, genera such as *Neocosmospora* and *Sarocladium* are more abundant in IR, highlighting their involvement in the infection process, whereas other genera like *Mortierella* and *Fusarium* decrease. These findings illustrate how infection alters the microbial community structure, promoting the proliferation of pathogenic taxa while suppressing non-pathogenic or beneficial microbes, likely contributing to disease symptoms and reduced plant health in infected stems.

**Figure 3:**
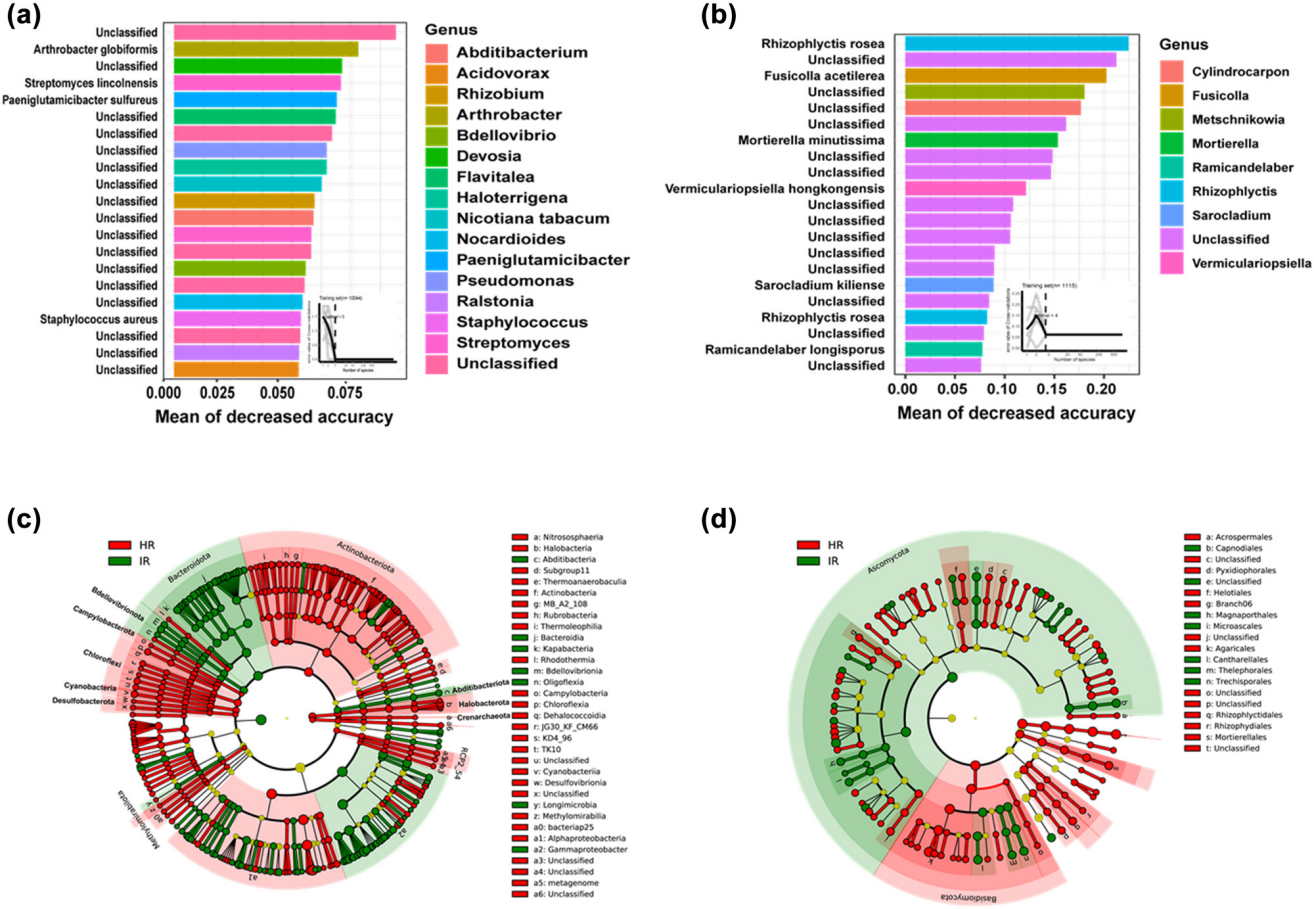
Biomarker analysis and phylogenetic distribution of microbial communities. (a) and (b) Top 21 bacterial and fungal zOTUs identified as biomarkers in healthy stem (HR) and infected stem (IR) using the Random Forest model. These biomarkers highlight significant differences in microbial communities between the two conditions. (c) and (d) Cladograms depicting the phylogenetic distribution of the most differentially abundant taxa in HR and IR for bacterial and fungal communities, respectively. Each circle’s diameter corresponds to the relative abundance of the taxa, with different colors indicating the most differentially abundant taxa. The circles represent phylogenetic levels from domain to genus, illustrating the hierarchical relationships and the shifts in community composition due to infection.

Further analysis using Random Forest and linear discriminant analysis effect size revealed significant differences in the microbial communities of HR and IR. In the bacterial communities (Figure 3(a)), key genera such as *Acidibacterium*, *Ralstonia*, and *Stenotrophomonas* were identified as important discriminators between HR and IR. Notably, *Ralstonia* exhibited higher importance in IR, underscoring its prominent role in the disease state. The fungal communities also displayed distinct differences, with genera such as *Cylindrocarpon*, *Neocosmospora*, and *Sarocladium* being more prominent in IR, further highlighting the shifts in microbial populations due to infection (Figure 3(b)). The cladograms provide a phylogenetic perspective, illustrating the differential abundance of bacterial and fungal taxa in HR and IR (Figure 3(c) and (d)). Red and green branches represent taxa significantly more abundant in IR and HR, respectively, visually demonstrating the impact of infection on microbial community structure. The enrichment of specific pathogenic taxa in infected stems likely contributes to disease progression and severity. Overall, these analyses highlight the profound changes in microbial communities associated with stem health, where infection leads to the dominance of pathogenic bacteria and fungi.

### 3.4 Infection-induced shifts in niche width

**Figure 4:**
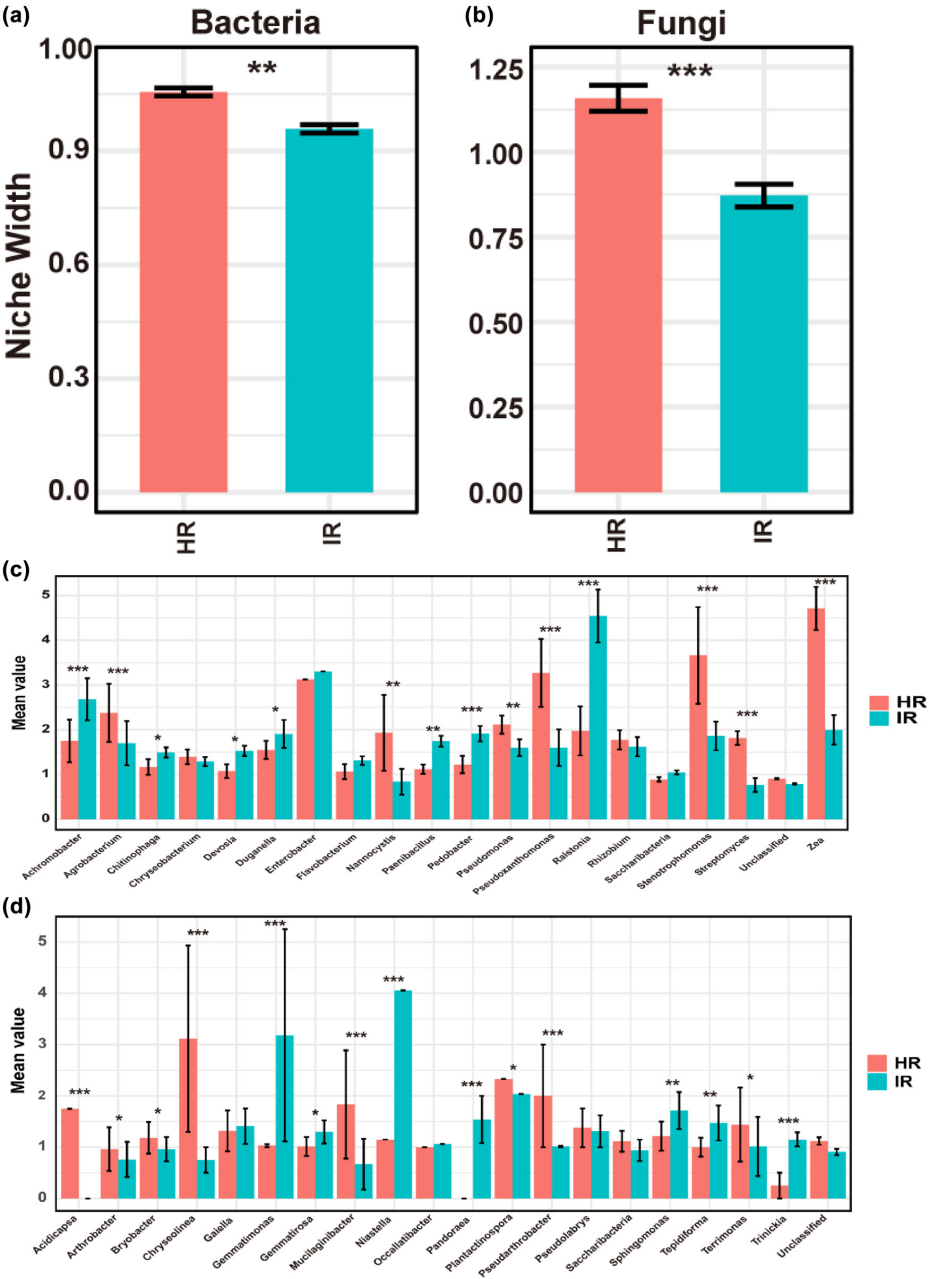
Niche breadth analysis of microbial communities. (a) Niche width of bacterial communities in healthy stem (HR) and infected stem (IR). The bar plot indicates a significant reduction in niche width for bacterial communities in IR compared to HR (*P* < 0.01**). (b) Niche width of fungal communities in HR and IR. The bar plot shows a significant decrease in niche width for fungal communities in IR compared to HR (*P* < 0.001***). (c) Mean values of relative abundance for various bacterial genera in HR and IR. Significant differences in abundance between HR and IR are indicated, highlighting the impact of infection on specific bacterial taxa (*P* < 0.05*, *P* < 0.01**, *P* < 0.001***). (d) Mean values of relative abundance for various fungal genera in HR and IR. The bar plot reveals significant changes in the abundance of certain fungal genera due to infection (*P* < 0.05*, *P* < 0.01**, *P* < 0.001***).

The analysis of niche width between healthy stems and infected stems reveals significant ecological shifts driven by infection. Bacterial communities in infected stems exhibit a markedly narrower niche width compared to those in healthy stems, with a statistically significant reduction (*P* < 0.01) (Figure 4a). This reduction suggests that bacterial communities in IR are confined to fewer ecological niches, indicating a decrease in overall diversity and potential functional redundancy. Similarly, fungal communities also show a substantial decrease in niche width in IR compared to HR (*P* < 0.001) (Figure 4b), highlighting the impact of infection in reducing ecological diversity within the fungal microbiome.

Further analysis of niche width at the genus level reveals additional insights into the ecological shifts caused by infection (Figure 4(c) and (d)). For example, in bacterial communities, genera such as *Ralstonia* show a significant increase in niche width in IR, indicating that these pathogens are adapting to and thriving within the narrower ecological niches imposed by disease conditions (Figure 4(c)). In contrast, the niche width of beneficial or neutral bacterial genera is reduced in IR, suggesting a constriction of their ecological roles within the infected environment. Similarly, in fungal communities, pathogenic genera like *Neocosmospora* demonstrate a wider niche width in IR, reinforcing the notion that infection fosters a more specialized and less diverse microbial community (Figure 4(d)). Collectively, these findings indicate that infection drives substantial reorganization of both bacterial and fungal communities, characterized by a loss of niche diversity and the rise of pathogenic taxa. This shift is likely a key factor contributing to the observed disease symptoms and underscores the critical role of microbial community dynamics in plant health and disease progression.

### 3.5 Infection-induced departure from neutrality in microbial community structure

**Figure 5:**
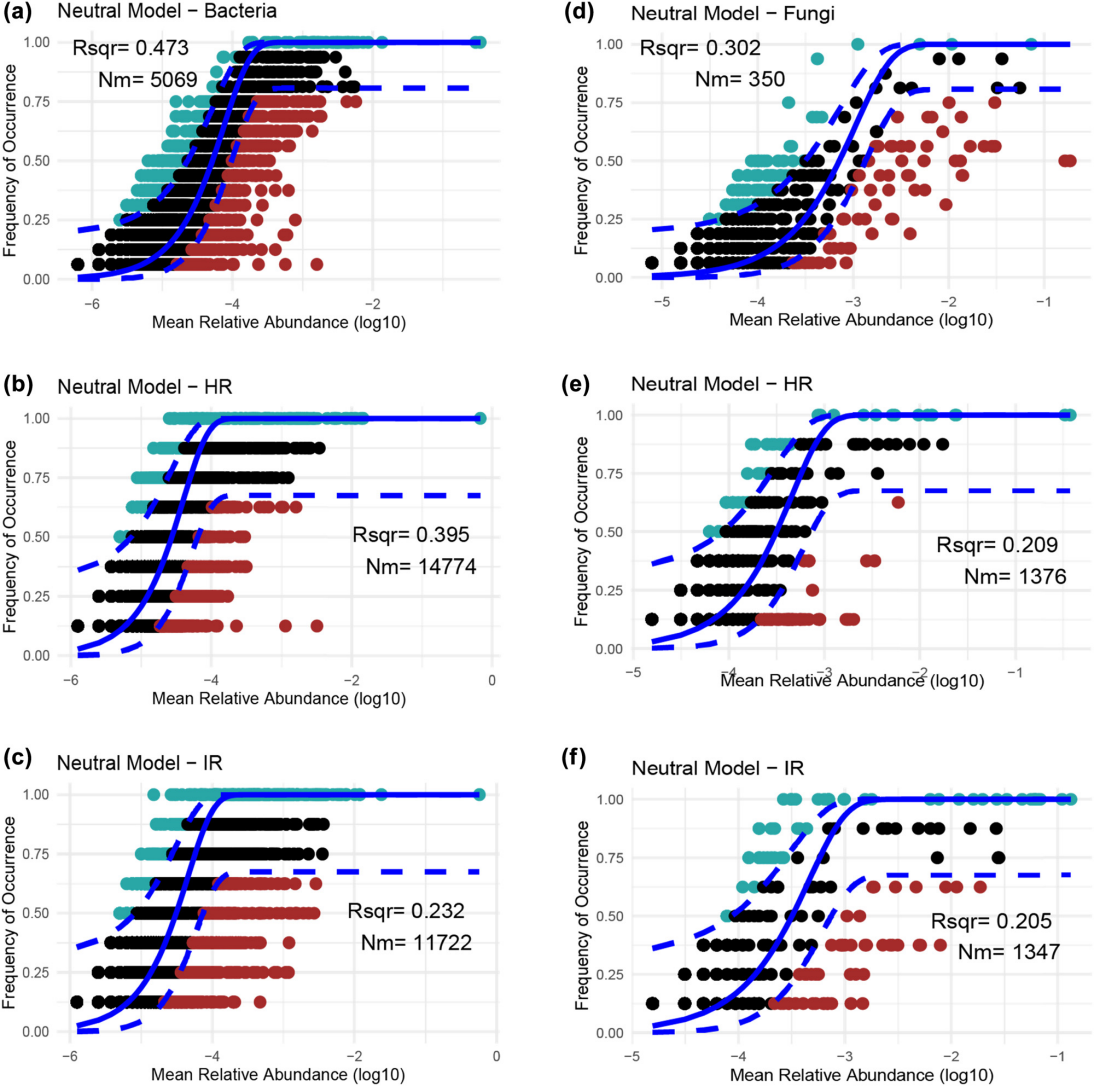
NST analysis for bacterial and fungal communities. (a) Overall fit of total bacterial communities to the neutral model. (b) and (c) Distribution of bacterial taxa in healthy stems (HR) and infected stems (IR), respectively. (d) Total fungal communities’ fit to the neutral model. (e) and (f) Alignment of fungal communities in HR and IR to the neutral model. *R*-squared represents the goodness-of-fit of the neutral model to the observed data, with higher values indicating a stronger influence of neutral processes. Nm denotes the immigration rate, or the number of individuals migrating into the community per generation, with higher values suggesting greater connectivity and dispersal within the community.

The neutral community model (NST) was applied to assess bacterial and fungal communities across different conditions, including all samples, healthy stems, and infected stems. The NST analysis revealed distinct patterns in the adherence of bacterial and fungal communities to neutral theory (Figure 5). Bacterial communities displayed a broader distribution of points (Figure 5(a)), indicating a stronger alignment with neutral theory, where a greater proportion of species exhibit behaviors consistent with neutral processes (*R* ^2^ = 0.437). In contrast, fungal communities showed a loose distribution (*R* ^2^ = 0.302), suggesting that while some species follow neutral theory, others may be influenced by additional non-neutral factors (Figure 5(d)). When focusing on healthy stems, bacterial communities continued to closely follow neutral patterns (*R* ^2^ = 0.395), reinforcing the notion that neutral processes predominantly shape these communities under healthy conditions (Figure 5(b)). However, fungal communities, though still largely neutral, exhibited slight deviations, implying that other ecological processes may also be influencing their composition in healthy stems (Figure 5(e)). In infected stems, a noticeable shift was observed. Both bacterial (*R* ^2^ = 0.232) and fungal (*R* ^2^ = 0.205) communities demonstrated significant departures from neutral theory, particularly within the fungal communities (Figure 5(c) and (f)). This deviation suggests that deterministic factors – such as the selective pressures exerted by infection – are becoming more influential, altering the microbial community structure in infected stems and reducing the role of neutral processes. Overall, these findings highlight the importance of neutral processes in structuring microbial communities in both healthy and infected stems. However, the presence of infection introduces deterministic forces that disrupt this balance, particularly in fungal communities, where infection drives a more pronounced departure from neutrality. This shift likely contributes to the observed differences in microbial community composition and function between healthy and infected stems.

### 3.6 Network analysis of microbial interactions reveals synergistic and antagonistic dynamics during pathogen co-infection in soil ecosystems

We utilized molecular ecological network (MEN) analysis to investigate the changes in microbial interactions following pathogen co-infection. To ensure comparability between different networks, we applied the same threshold values (0.91 for bacterial communities and 0.85 for fungal communities) when constructing MENs for both healthy and infected soil samples. The overall topological indices revealed that the average path lengths (GD) of all networks ranged from 3.898 to 11.219. These values are close to the logarithm of the total number of network nodes and exceed those of the corresponding random networks (Tables S3 and S4), indicating that the MENs exhibit typical small-world network characteristics. The modularity of the networks, ranging from 0.588 to 0.924 for both bacterial and fungal communities, was also significantly higher than that of their respective random networks, suggesting that all constructed networks possess a modular topology. These critical topological features enabled us to proceed with further analysis of the networks.

Further analysis revealed significant differences in the complexity and connectivity of microbial interactions under healthy versus diseased conditions. The ecological networks of HR and IR showed that healthy stems had more interconnected and complex network structures, with numerous nodes and links representing interactions between bacterial and fungal groups (Tables S5 and S6). In contrast, networks in infected stems were notably sparser, with fewer connections and more isolated nodes, indicating a disruption in microbial interactions due to the infection. *Z*_*i*_–*P*_*i*_ analysis results indicated that the number of keystone species in the bacterial community network was lower in healthy soil (2 nodes) compared to infected soil (3 nodes). However, for the fungal community network, the number of keystone species was higher in healthy soil (20 nodes) than in diseased soil (1 node) (Figure S5). Network stability analysis showed a significant decrease in the robustness of endophytic bacterial and fungal communities after infection (*P* < 0.001), with bacterial community robustness decreasing from 0.373 to 0.353 and fungal community robustness decreasing from 0.433 to 0.341 (Figure S6(a)). Additionally, infection markedly increased network vulnerability, rising from 0.121 and 0.027 to 0.249 and 0.287, respectively (Figure S6(b)). This suggests that co-infection disrupted the original defense systems of the network, reducing the stability of the endophytic communities.

**Figure 6:**
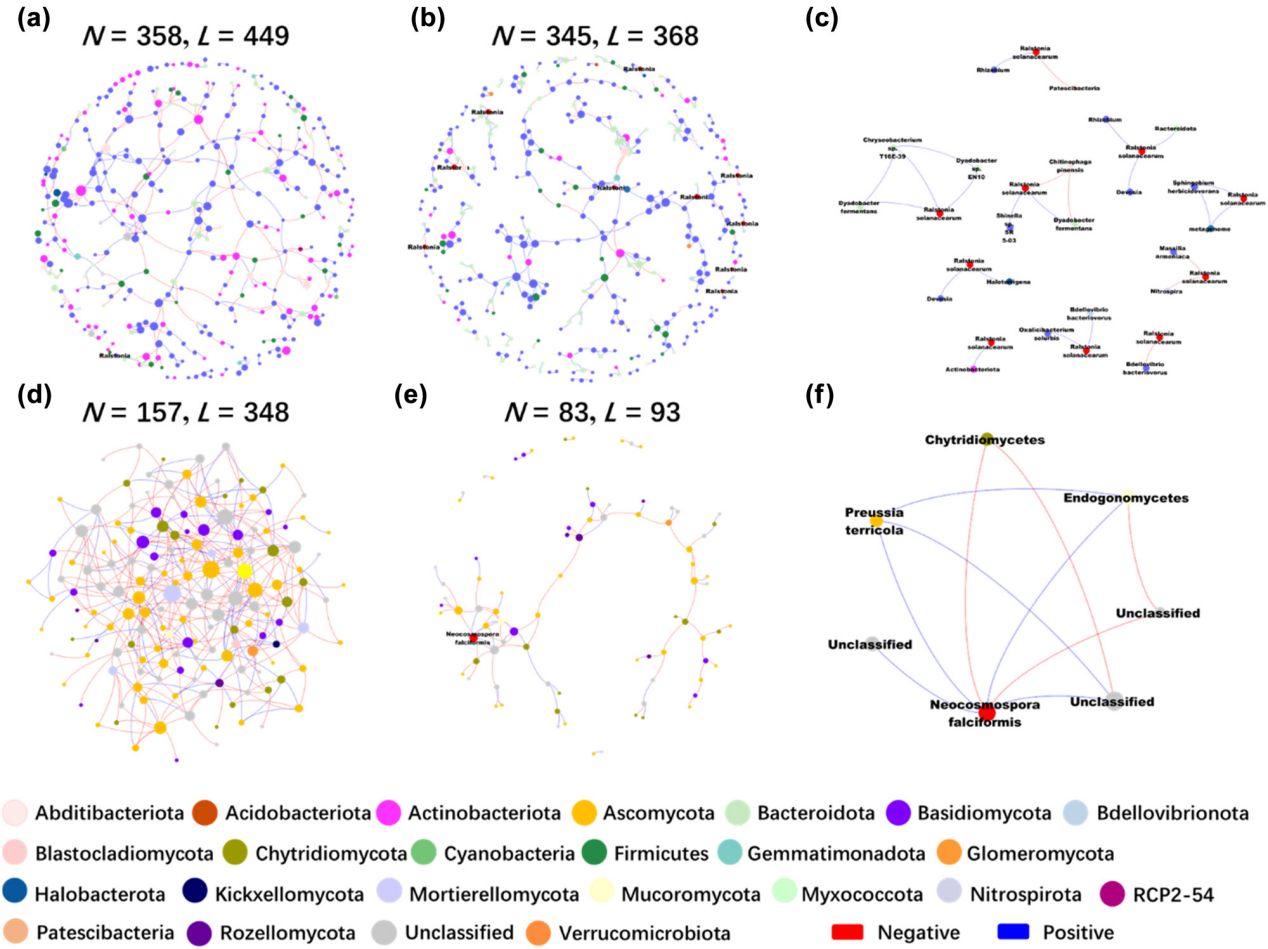
Intra-domain network analysis of bacterial and fungal communities. (a) Bacterial network of healthy samples. (b) Bacterial network of infected samples. (c) Bacterial sub-network of infected samples. (d) Fungal network of healthy samples. (e) Fungal network of infected samples. (f) Fungal sub-network of infected samples. Different colors represent distinct phyla within the bacterial and fungal communities. The size of each node reflects its node degree, indicating the number of direct connections it has within the network. In all networks, red links denote negative correlations between nodes, which may indicate competitive or antagonistic interactions.

To gain deeper insights into the dynamics of co-infection, we focused on the pathogen-associated subnetworks – comprising nodes directly linked to pathogens within the broader network. This approach allowed us to explore the interactions between pathogens and antagonistic microbiomes, which may play critical roles in modulating the microbial community’s response to infection. Our analysis revealed that *R. solanacearum*, a known pathogen, exhibited positive correlations with a diverse set of bacteria, including *Rhizobium*, *Dyadobacter*, *Bdellovibrio*, *Chryseobacterium*, *Devosia*, *Haloterrigena*, *Oxalicibacterium*, and *Sphingobium* (Figure 6(c)). These positive associations suggest potential cooperative or synergistic interactions that could influence the persistence and virulence of *Ralstonia* in the soil environment. Conversely, *Ralstonia* showed negative correlations with *Bdellovibrio bacteriovorus*, *Chitinophaga pinensis*, *Brevundimonas*, *Massilia*, and *Nitrospira*, indicating potential antagonistic interactions that might inhibit its proliferation or pathogenicity. Similarly, within the fungal community, *N. falciformis*, a pathogenic fungus, was positively correlated with *Preussia terricola* and *Endogonomycetes*, suggesting possible cooperative relationships that could enhance its survival or pathogenicity (Figure 6(f)). On the other hand, *Neocosmospora* exhibited a negative correlation with *Chytridiomycetes*, which may represent an antagonistic interaction, potentially limiting its impact on the host. These findings highlight the complex interplay between pathogens and other microbial species during co-infection, revealing both synergistic and antagonistic relationships that could significantly influence disease outcomes.

### 3.7 Synergistic and antagonistic relationships in fungal–bacterial co-infections

**Figure 7:**
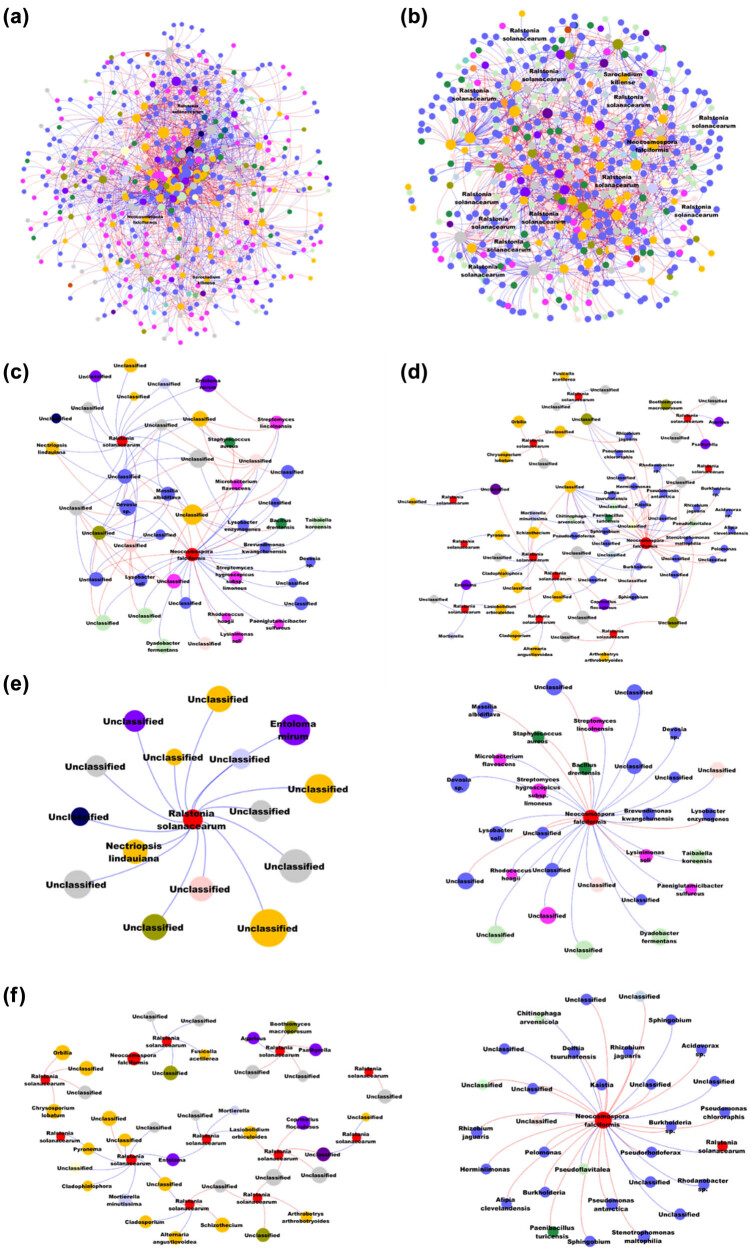
Interdomain ecological networks of bacterial–fungal associations in healthy and infected samples. (a) Interdomain ecological network illustrating bacterial–fungal associations in healthy stem samples (HR). This network represents the overall interactions between bacterial and fungal communities in a healthy state. (b) Interdomain ecological network of bacterial–fungal associations in infected stem samples (IR), showing how these interactions are altered in the presence of infection. (c) Sub-network focusing on pathogenic microorganisms within the healthy stem samples, highlighting the interactions specific to pathogenic taxa like *R. solanacearum* and *N. falciformi*. (d) Sub-network of pathogenic microorganisms in the infected stem samples, detailing how the interactions between these pathogens and other microbial taxa change under infection conditions. (e) Targeted sub-network in healthy stem samples, displaying only the interactions between each pathogenic microorganism (*R. solanacearum* and *N. falciformis*) and their associated nodes. (f) Targeted sub-network in infected stem samples, showing the interactions of each pathogenic microorganism (*R. solanacearum* and *N. falciformis*) and their associated nodes under infection.

The bipartite network analysis of fungal and bacterial interactions reveals the intricate and complex relationships between pathogens and their associated microbial communities under co-infection conditions (Figure 7(a) and (b)). *R. solanacearum* demonstrates both direct and indirect associations with various fungal species, suggesting that this pathogen may manipulate the surrounding microbial community to create an environment more conducive to its proliferation, thereby promoting disease progression (Figure 7(c) and (d)). Specifically, *Chrysosporium lobatum*, *Psathyrella* spp., *Orbilia* spp., *Fusicolla acetilerea*, *N. falciformis* and *Cladophialophora* spp. are directly associated with *R. solanacearum*, indicating their potential roles as facilitators in the bacterial invasion process (Figure 7(e) and (f)). These fungi may either weaken the host plant’s defenses or engage in synergistic interactions that enhance the bacterium’s virulence. Conversely, other fungi such as *Alternaria angustivoidea*, *Schizothecium* spp., *Coprinellus flocculosus*, and *Arthrobotrys arthrobotryoides* have been identified as possible antagonists. These species may inhibit *R. solanacearum* by competing for resources, producing antimicrobial compounds, or altering microbial community dynamics in a way that reduces the bacterium’s ability to establish infection. The network analysis also reveals *N. falciformis* with a diverse array of bacterial species (Figure 7(e) and (f)). The network analysis centered on *N. falciformis* highlights its extensive interactions with a wide range of bacterial species, suggesting its significant role in shaping the microbial community associated with tobacco plants. *N. falciformis* is closely linked to several bacterial taxa, including *Pseudomonas chlororaphis*, *Burkholderia* sp., *Stenotrophomonas maltophilia*, and *Rhizobium jaguaris*, which may serve as facilitators in its invasion and pathogenicity. Conversely, the network also identifies bacteria such as *Pseudorhodofrax* spp. and *Sphingobium* spp. Additionally, we identified a direct interaction between *R. solanacearum* and *N. falciformis* (Figure 7(f)), providing compelling evidence of synergistic pathogen cooperation under co-infection conditions. This direct relationship suggests that these two pathogens may collaborate to enhance their invasion efficiency, potentially by jointly manipulating the host’s immune responses or by creating a more favorable environment for mutual survival and proliferation.

## 4 Discussion

This study sought to unravel the complex interactions between bacterial and fungal pathogens in tobacco plants, with a focus on the co-infection dynamics involving *Ralstonia* and *N. falciformis*. Our objectives were aimed at addressing gaps in our understanding of how these pathogens interact with each other and with the broader microbial community, as well as the ecological shifts induced by infection. By achieving these objectives, we aimed to contribute to the development of targeted disease management strategies that leverage both the synergistic and antagonistic relationships within microbial communities. While prior studies have explored the roles of individual pathogens in disease progression, the interplay between multiple pathogens and their collective impact on disease outcomes remains underexplored [18,20]. A comprehensive review of the literature reveals a lack of studies that fully address the cooperative and competitive interactions between different microbial taxa in the context of co-infection [16]. A key mechanism driving the shifts in microbial diversity observed in this study is the altered physiological environment within infected plant tissues. Although the effects of pathogens like *R. solanacearum* on plant health are well documented [20,47], the intricate network of relationships involving co-infecting fungi and bacteria, and their influence on disease severity, has not been adequately investigated.

The study revealed significant alterations in microbial community structure and diversity between healthy and infected tobacco stems (Figure S4, Tables S1 and S2), highlighting the profound impact of infection on the microbial ecosystem associated with plant health. A key observation was the reduction in microbial diversity within infected stems (Figure S2), which has critical implications for the resilience and stability of the plant’s microbiome. The reduction in diversity can be attributed to competitive exclusion, where pathogenic taxa, due to their metabolic and ecological advantages, outcompete beneficial microbes for limited resources [34]. In healthy stems, a diverse microbial community typically provides a robust network of interactions that buffer against environmental stresses and pathogenic invasions [48,49]. However, infection disrupts this balance, leading to a marked decrease in diversity, particularly within the fungal community [7,50]. The dominance of *R. solanacearum* and *N. falciformis* in infected stems could also reflect a synergistic relationship between the pathogens, wherein each pathogen alters the plant’s internal environment to favor the other’s growth. For example, *N. falciformis* may lower the oxygen levels or change pH, creating a more favorable environment for *Ralstonia* [51]. This environmental modification is a key mechanism behind the observed niche specialization, where pathogens thrive in newly altered microenvironments while other microbes are excluded. This reduction in diversity is concerning as it suggests a loss of functional redundancy within the microbiome [52], where fewer microbial species are available to perform essential ecological roles [53]. Consequently, the plant’s overall resilience to additional biotic or abiotic stresses may be compromised, making it more susceptible to further infections or environmental fluctuations [54,55]. The dominance of pathogenic taxa, such as *R. solanacearum* and *N. falciformis*, in infected stems is a central factor driving these changes in community structure (Figure 1). These pathogens not only increase in abundance but also alter the composition of the surrounding microbial community (Figure 2). The shift toward a pathogen-dominated community can lead to a less resilient ecosystem, where the loss of beneficial or neutral microbial species further destabilizes the microbial network [34]. This dominance of pathogens creates an environment more conducive to disease progression, as the interactions between the remaining microbial species may be less complex and less capable of providing the same level of ecosystem services as seen in healthy stems [56,57]. For example, beneficial microbes that typically contribute to nutrient cycling, disease suppression, or plant growth promotion may be outcompeted or suppressed, leading to a decline in overall plant health [58,59].

The analysis of niche width provides additional insights into how infection drives specialization among pathogens and reduces ecological diversity [60]. In healthy stems, a broader niche width among microbial species reflects a more diverse range of ecological roles and interactions, allowing the community to respond flexibly to changes in environmental conditions or the presence of pathogens [61,62]. However, in infected stems, the narrowing of niche width indicates that the microbial community is becoming more specialized [63,64]. Pathogens like *R. solanacearum* and *N. falciformis* appear to be adapting to the specific conditions created by infection, such as altered nutrient availability or immune suppression, which allows them to thrive while other species are excluded [35,65]. This specialization reduces the overall ecological diversity within the microbial community, potentially making it more vulnerable to further disturbances [66]. The observed patterns in microbial community dynamics align with findings from other studies on infected plants, which often report a similar reduction in microbial diversity and a shift toward pathogen-dominated communities [6,50]. These patterns can be attributed to several factors. First, the immune response of the plant to infection may selectively suppress certain microbial species while allowing others, particularly pathogens, to proliferate [67]. Second, the altered physical and chemical environment within the infected tissues may create conditions that are more favorable to specific pathogens, thereby reducing the ecological niches available to other species [68]. Finally, the interaction between co-infecting pathogens may further drive the exclusion of non-pathogenic species, as these pathogens may modify the environment in ways that are beneficial to their own survival but detrimental to other microbes [69,70].

Our analysis of microbial community assembly under infected conditions revealed a significant deviation from neutral theory, indicating a shift toward deterministic processes in both bacterial and fungal communities (Figure 4). Neutral theory posits that the structure of ecological communities is largely shaped by stochastic processes such as random dispersal, demographic fluctuations, and speciation, with all species having an equal chance of survival and reproduction [71,72]. However, the marked departure from neutrality observed in this study suggests that under infection, deterministic factors – particularly selective pressures exerted by pathogens – play a more dominant role in shaping microbial community composition and structure (Figure 4(a) and (b)). In healthy plant tissues, microbial communities are often structured by a combination of neutral and deterministic processes, maintaining a balance that allows for both diversity and stability [38]. However, the onset of infection introduces strong selective pressures that disrupt this balance [50]. The presence of aggressive pathogens such as *R. solanacearum* and *N. falciformis* likely imposes specific environmental conditions that favor certain microbial taxa over others [18]. These conditions might include changes in nutrient availability, pH, oxygen levels, and the presence of toxic compounds produced by the pathogens or by the plant in response to infection [73]. As a result, microbial taxa that can tolerate or even thrive under these stressors are selected for, leading to a community structure that is increasingly driven by deterministic processes rather than neutral ones.

The ecological implications of deterministic processes are profound. A microbial community shaped by deterministic forces is likely to exhibit reduced resilience to environmental fluctuations, as fewer taxa occupy a narrower range of niches. This reduced functional redundancy can make the community more vulnerable to additional disturbances or stresses, such as pathogen invasions, climate shifts, or changes in host physiology [74]. While neutral theory suggests that the loss of one species in a diverse community can be mitigated by others, our findings indicate that in infected plant tissues, the dominance of specific taxa can lead to more severe ecological consequences if those taxa are lost or outcompeted [75,76]. In other words, the loss of a few specialized taxa in a deterministic community might disproportionately impact the community’s functionality, increasing susceptibility to disease and instability. This finding aligns with studies showing that pathogen-induced selective pressures can drive microbial communities toward specialization and reduced biodiversity [77]. It also challenges traditional views of microbial communities as primarily shaped by stochastic processes, highlighting the importance of environmental and biological factors that strongly select for certain community members [78]. The role of deterministic factors is further supported by the observed reduction in microbial diversity and niche width (Figure S2, Figure 4). As dominant taxa outcompete others for limited resources, they reinforce their position within the community, further reducing diversity and restricting ecological niches [78,79]. Consequently, the community becomes more specialized, with fewer taxa fulfilling defined roles in the ecosystem. A community structured by deterministic processes is less resilient to changes and stresses [80]. In contrast, a neutral community with multiple species playing overlapping roles offers a buffer against disturbances, as the loss of one species can be compensated for by others [75,76]. In deterministic communities, however, the loss of even a single species may have a significant impact on stability and functionality [74], making the microbial ecosystem in infected plant tissues more vulnerable to further infections or environmental stressors, potentially exacerbating disease progression.

Comparing these findings with other studies, it is clear that the balance between neutral and deterministic processes in plant-associated microbial communities can vary widely depending on the specific context [46,81]. In some cases, such as in healthy or undisturbed environments, neutral processes may play a more significant role, leading to higher diversity and greater functional redundancy [38]. However, in stressed or infected environments, the influence of deterministic factors often becomes more pronounced, leading to the kind of community shifts observed in this study. Similar deviations from neutrality have been documented in other plant-pathogen systems, where infection leads to a more deterministic assembly of microbial communities, often characterized by a decline in diversity and an increase in the dominance of certain pathogenic or opportunistic taxa [82,83]. Overall, the deviation from neutrality observed in this study underscores the importance of deterministic processes in shaping microbial community structure under infection. This shift toward deterministic assembly has critical implications for the stability and functionality of the microbial ecosystem within infected plant tissues.

Network analysis was always used to explore the interaction between microbiomes [29]. Our results revealed complex synergistic relationships between *R. solanacearum* and *N. falciformis* (Figures 6 and 7). These interactions suggest a coordinated effort among these pathogens to establish and exacerbate infections in tobacco plants. One plausible mechanism for this synergy could be resource sharing, where the pathogens alter the plant’s physiology, making essential nutrients more available to all co-infecting pathogens [84,85]. For example, the degradation of plant cell walls by fungal enzymes might release sugars [86,87] and other metabolites that *R. solanacearum* can utilize, thereby enhancing its proliferation. Additionally, these fungi might suppress the plant’s immune responses through the production of secondary metabolites that either directly inhibit plant defense mechanisms or modulate the plant’s signaling pathways, creating a more conducive environment for bacterial invasion [88,89]. Environmental modification is another potential mechanism underlying these synergistic interactions [90,91]. *N. falciformis* could alter the microenvironment within the plant tissues, such as by changing pH levels or oxygen availability [19,23], which could favor the growth and virulence of *R. solanacearum*. This environmental modification could also involve the creation of biofilms, which are known to protect bacterial pathogens from both the host immune system and external treatments, thereby facilitating a more persistent infection [92]. On the other hand, the study also identified antagonistic relationships within the microbial community, where certain taxa appear to inhibit the growth or virulence of *R. solanacearum* and associated fungi. Notably, bacteria such as *B. bacteriovorus* and *C. pinensis* were found to exhibit negative correlations with these pathogens, suggesting their potential role as natural antagonists. *B. bacteriovorus*, a predatory bacterium, could reduce *R. solanacearum* populations by directly preying on them [93,94], while *C. pinensis* might inhibit fungal pathogens through the degradation of chitin, a major component of fungal cell walls [95]. These antagonistic interactions are crucial because they provide a natural counterbalance to the spread of infection, potentially preventing the complete dominance of pathogenic species within the microbial community. Interestingly, we also found a positive correlation between *Rhizobium* and *R. solanacearum* during co-infection, consistent with previous research [6]. This suggests that Rhizobium may act as a “traitor” during co-infection, assisting the pathogen in its invasion and contributing to the development of tobacco disease. These findings highlight the complex interplay between synergistic and antagonistic forces in microbial communities, offering valuable insights for developing targeted strategies to manage plant diseases.

## 5 Conclusion

This study provides a novel contribution to understanding microbial community dynamics under co-infection conditions, with a particular focus on the deterministic processes driving community assembly in plant stems. Our findings highlight that co-infection by *R.* solanacearum and *N. falciformis* induces significant shifts in microbial structure and diversity, predominantly driven by deterministic processes rather than stochastic ones. We observed that infection not only reduces microbial diversity but also narrows the ecological niche, leading to greater specialization and reduced resilience within the microbial community. This ecological specialization is a key feature of microbial community assembly under disease pressure, where pathogens such as *R. solanacearum* exploit specific conditions within infected tissues, thereby reshaping the microbial landscape. Furthermore, the network analyses reveal complex interactions between pathogens and other microbial species, identifying both synergistic and antagonistic relationships that influence disease progression. These findings have important implications for disease management, as they suggest that manipulating microbial communities could enhance plant health. Promoting beneficial microbes or restoring functional redundancy within the plant microbiome may help mitigate disease severity by buffering the negative impacts of pathogenic dominance. Furthermore, interventions aimed at modulating the microenvironment created by pathogens, could help reduce the success of co-infecting pathogens. However, it is important to note that potential biases may arise from the sequencing approach used in this study, including issues related to the resolution of certain microbial taxa or biases in DNA extraction and amplification. Additionally, the use of tobacco as a model system may not fully represent microbial community dynamics in other plant species, limiting the generalizability of some findings. This study underscores the critical importance of considering microbial interactions when developing strategies for managing plant diseases. Given the pivotal role of deterministic processes in shaping microbial communities, future research should focus on identifying specific microbial interactions that can be targeted to enhance plant resilience. Additionally, exploring interventions that can manipulate microbial communities, such as microbial inoculants or tailored microbiome-based strategies, holds promise for improving plant health. By better understanding the complex relationships between microbial diversity, community structure, and disease dynamics, future studies may provide actionable insights into more sustainable and targeted disease management approaches.

## Supplementary Material

Supplementary material

## References

[1] Sierro N, Battey JND, Ouadi S, Bakaher N, Bovet L, Willig A, et al. The tobacco genome sequence and its comparison with those of tomato and potato. Nat Commun. 2014;5(1):3833. 10.1038/ncomms4833 PMC4024737 24807620

[2] Tang Z, Chen L, Chen Z, Fu Y, Sun X, Wang B, et al. Climatic factors determine the yield and quality of Honghe flue-cured tobacco. Sci Rep. 2020;10(1):19868. 10.1038/s41598-020-76919-0 PMC7669845 33199769

[3] Warner KE, Fulton GA. Importance of tobacco to a country’s economy: an appraisal of the tobacco industry’s economic argument. Tob Control. 1995;4(2):180–3.

[4] DeCicca P, Kenkel D, Lovenheim MF. The economics of tobacco regulation: a comprehensive review. J Econ Lit. 2022;60(3):883–970. 10.1257/jel.20201482 PMC10072869 37075070

[5] Peruga A, López MJ, Martinez C, Fernández E. Tobacco control policies in the 21st century: achievements and open challenges. Mol Oncol. 2021;15(3):744–52. 10.1002/1878-0261.12918 PMC7931122 33533185

[6] Hu Q, Tan L, Gu S, Xiao Y, Xiong X, Zeng W-A, et al. Network analysis infers the wilt pathogen invasion associated with non-detrimental bacteria. NPJ Biofilms Microbiomes. 2020;6(1):8. 10.1038/s41522-020-0117-2 PMC7021801 32060424

[7] Tan L, Xiao Y, Zeng W-A, Gu S, Zhai Z, Wu S, et al. Network analysis reveals the root endophytic fungi associated with Fusarium root rot invasion. Appl Soil Ecol. 2022;178:104567.

[8] Oerke EC. Crop losses to pests. J Agric Sci. 2006;144(1):31–43.

[9] He H, Pan Z, Wu J, Hu C, Bai L, Lyu J. Health effects of tobacco at the global, regional, and national levels: results from the 2019 global burden of disease study. Nicotine Tob Res. 2021;24(6):864–70. 10.1093/ntr/ntab265 34928373

[10] Ke X, Chen J, Zuo C, Wang X. The cropland intensive utilisation transition in China: an induced factor substitution perspective. Land Use Policy. 2024;141:107128.

[11] Liu CLC, Kuchma O, Krutovsky KV. Mixed-species versus monocultures in plantation forestry: development, benefits, ecosystem services and perspectives for the future. Glob Ecol Conserv. 2018;15:e00419.

[12] Dudenhöffer J-H, Luecke NC, Crawford KM. Changes in precipitation patterns can destabilize plant species coexistence via changes in plant–soil feedback. Nat Ecol Evol. 2022;6(5):546–54. 10.1038/s41559-022-01700-7 35347257

[13] Singh BK, Delgado-Baquerizo M, Egidi E, Guirado E, Leach JE, Liu H, et al. Climate change impacts on plant pathogens, food security and paths forward. Nat Rev Microbiol. 2023;21(10):640–56. 10.1038/s41579-023-00900-7 PMC10153038 37131070

[14] Karvonen A, Jokela J, Laine A-L. Importance of sequence and timing in parasite coinfections. Trends Parasitol. 2019;35(2):109–18. 10.1016/j.pt.2018.11.007 30578150

[15] Sabey KA, Song SJ, Jolles A, Knight R, Ezenwa VO. Coinfection and infection duration shape how pathogens affect the African buffalo gut microbiota. ISME J. 2021;15(5):1359–71. 10.1038/s41396-020-00855-0 PMC8115229 33328653

[16] Devi P, Khan A, Chattopadhyay P, Mehta P, Sahni S, Sharma S, et al. Co-infections as modulators of disease outcome: minor players or major players? Front Microbiol. 2021;12:664386. 10.3389/fmicb.2021.664386 PMC8290219 34295314

[17] Graham AL, Cattadori IM, Lloyd-Smith JO, Ferrari MJ, Bjørnstad ON. Transmission consequences of coinfection: cytokines writ large? Trends Parasitol. 2007;23(6):284–91. 10.1016/j.pt.2007.04.005 17466597

[18] Mansfield J, Genin S, Magori S, Citovsky B, Sriariyanum M, Ronald P, et al. Top 10 plant pathogenic bacteria in molecular plant pathology. Mol Plant Pathol. 2012;13(6):614–29. 10.1111/j.1364-3703.2012.00804.x PMC6638704 22672649

[19] Sáenz V, Lizcano Salas AF, Gené J, Celis Ramírez AM. Fusarium and Neocosmospora: fungal priority pathogens in laboratory diagnosis. Crit Rev Microbiol. 2024;1–14. 10.1080/1040841X.2024.2369693 38949272

[20] Ahmed W, Yang J, Tan Y, Munir S, Liu Q, Zhang J, et al. Ralstonia solanacearum, a deadly pathogen: revisiting the bacterial wilt biocontrol practices in tobacco and other Solanaceae. Rhizosphere. 2022;21:100479.

[21] An Y, Zhang M. Advances in understanding the plant-Ralstonia solanacearum interactions: unraveling the dynamics, mechanisms, and implications for crop disease resistance. N Crop. 2024;1:100014.

[22] González V, García-Martínez S, Ruiz JJ, Flores-León A, Picó B, Garcés-Claver A. First report of Neocosmospora falciformis causing wilt and root rot of muskmelon in Spain. Plant Dis. 2020;104(4):1256.

[23] Xu C, Ding D, He X, Liu B. First report of Sarocladium kiliense causing brown rot of Hypsizygus marmoreus in China. Plant Dis. 2022;106(7):1990.

[24] Okon EM, Okocha RC, Taiwo AB, Michael FB, Bolanle AM. Dynamics of co-infection in fish: a review of pathogen-host interaction and clinical outcome. Fish Shellfish Immunol Rep. 2023;4:100096. 10.1016/j.fsirep.2023.100096 PMC10213192 37250211

[25] Semenec L, Cain AK, Dawson CJ, Liu Q, Dinh H, Lott H, et al. Cross-protection and cross-feeding between Klebsiella pneumoniae and Acinetobacter baumannii promotes their co-existence. Nat Commun. 2023;14(1):702. 10.1038/s41467-023-36252-2 PMC9911699 36759602

[26] Abdullah AS, Moffat CS, Lopez-Ruiz FJ, Gibberd MR, Hamblin J, Zerihun A. Host-multi-pathogen warfare: pathogen interactions in co-infected plants. Front Plant Sci. 2017;8:1806. 10.3389/fpls.2017.01806 PMC5660990 29118773

[27] Lalbiaktluangi C, Yadav MK, Singh PK, Singh A, Iyer M, Vellingiri B, et al. A cooperativity between virus and bacteria during respiratory infections. Front Microbiol. 2023;14:1279159. 10.3389/fmicb.2023.1279159 PMC10720647 38098657

[28] Hassan A, Blanchard N. Microbial (co)infections: powerful immune influencers. PLOS Pathog. 2022;18(2):e1010212. 10.1371/journal.ppat.1010212 PMC8812865 35113966

[29] Deng Y, Jiang Y-H, Yang Y, He Z, Luo F, Zhou J. Molecular ecological network analyses. BMC Bioinf. 2012;13(1):113. 10.1186/1471-2105-13-113 PMC3428680 22646978

[30] Zheng H, Yang T, Bao Y, He P, Yang K, Mei X, et al. Network analysis and subsequent culturing reveal keystone taxa involved in microbial litter decomposition dynamics. Soil Biol Biochem. 2021;157:108230.

[31] Feng K, Zhang Z, Cai W, Liu W, Xu M, Yin H, et al. Biodiversity and species competition regulate the resilience of microbial biofilm community. Mol Ecol. 2017;26(21):6170–82. 10.1111/mec.14356 28926148

[32] Waheed A, Haxim Y, Islam W, Kahar G, Liu X, Zhang D. Role of pathogen’s effectors in understanding host–pathogen interaction. Biochim Biophys Acta – Mol Cell Res. 2022;1869(12):119347. 10.1016/j.bbamcr.2022.119347 36055522

[33] Liu H, Li J, Carvalhais LC, Percy CD, Prakash Verma J, Schenk PM, et al. Evidence for the plant recruitment of beneficial microbes to suppress soil-borne pathogens. N Phytol. 2021;229(5):2873–85. 10.1111/nph.17057 33131088

[34] Trivedi P, Leach JE, Tringe SG, Sa T, Singh BK. Plant–microbiome interactions: from community assembly to plant health. Nat Rev Microbiol. 2020;18(11):607–21. 10.1038/s41579-020-0412-1 32788714

[35] Mallon CA, Poly F, Le Roux X, Marring I, van Elsas JD, Salles JF. Resource pulses can alleviate the biodiversity-invasion relationship in soil microbial communities. Ecology. 2015;96(4):915–26. 10.1890/14-1001.1 26230013

[36] Guo Y, Luo H, Wang L, Xu M, Wan Y, Chou M, et al. Multifunctionality and microbial communities in agricultural soils regulate the dynamics of a soil-borne pathogen. Plant Soil. 2021;461(1):309–22.

[37] Liu W, Zhang Z, Wan S. Predominant role of water in regulating soil and microbial respiration and their responses to climate change in a semiarid grassland. Glob Change Biol. 2009;15(1):184–95.

[38] Gu S, Xiong X, Tan L, Deng Y, Du X, Yang X, et al. Soil microbial community assembly and stability are associated with potato (Solanum tuberosum L.) fitness under continuous cropping regime. Front Plant Sci. 2022;13:1000045. 10.3389/fpls.2022.1000045 PMC9574259 36262646

[39] Frank DN. BARCRAWL and BARTAB: software tools for the design and implementation of barcoded primers for highly multiplexed DNA sequencing. BMC Bioinf. 2009;10(1):362. 10.1186/1471-2105-10-362 PMC2777893 19874596

[40] Kong Y. Btrim: a fast, lightweight adapter and quality trimming program for next-generation sequencing technologies. Genomics. 2011;98(2):152–3. 10.1016/j.ygeno.2011.05.009 21651976

[41] DeSantis TZ, Hugenholtz P, Larsen N, Rojas M, Brodie EL, Keller K, et al. Greengenes, a chimera-checked 16S rRNA gene database and workbench compatible with ARB. Appl Environ Microbiol. 2006;72(7):5069–72. 10.1128/AEM.03006-05 PMC1489311 16820507

[42] Nilsson RH, Tedersoo L, Ryberg M, Kristiansson E, Hartmann M, Unterseher M, et al. A comprehensive, automatically updated fungal ITS sequence dataset for reference-based chimera control in environmental sequencing efforts. Microbes Environ. 2015;30(2):145–50. 10.1264/jsme2.ME14121 PMC4462924 25786896

[43] Feng K, Peng X, Zhang Z, Gu S, He Q, Shen W, et al. iNAP: an integrated network analysis pipeline for microbiome studies. iMeta. 2022;1(2):e13. 10.1002/imt2.13 PMC10989900 38868563

[44] Bascompte J, Jordano P, Melián CJ, Olesen JM. The nested assembly of plant–animal mutualistic networks. Proc Natl Acad Sci. 2003;100(16):9383–7. 10.1073/pnas.1633576100 PMC170927 12881488

[45] Logares R, Lindström ES, Langenheder S, Logue JB, Paterson H, Laybourn-Parry J, et al. Biogeography of bacterial communities exposed to progressive long-term environmental change. ISME J. 2013;7(5):937–48. 10.1038/ismej.2012.168 PMC3635229 23254515

[46] Jiao S, Yang Y, Xu Y, Zhang J, Lu Y. Balance between community assembly processes mediates species coexistence in agricultural soil microbiomes across eastern China. ISME J. 2020;14(1):202–16. 10.1038/s41396-019-0522-9 PMC6908645 31611655

[47] Li M, Pommier T, Yin Y, Wang J, Gu S, Jousset A, et al. Indirect reduction of Ralstonia solanacearum via pathogen helper inhibition. ISME J. 2022;16(3):868–75. 10.1038/s41396-021-01126-2 PMC8857195 34671104

[48] Konopka A, Lindemann S, Fredrickson J. Dynamics in microbial communities: unraveling mechanisms to identify principles. ISME J. 2015;9(7):1488–95. 10.1038/ismej.2014.251 PMC4478703 25526370

[49] Ji B, Herrgård MJ, Nielsen J. Microbial community dynamics revisited. Nat Comput Sci. 2021;1(10):640–1. 10.1038/s43588-021-00144-6 38217193

[50] Li P, Gu S, Zhu Y, Xu T, Yang Y, Wang Z, et al. Soil microbiota plays a key regulatory role in the outbreak of tobacco root rot. Front Microbiol. 2023;14:1214167. 10.3389/fmicb.2023.1214167 PMC10540700 37779693

[51] Sokolova GD, Budynkov NI, Tselipanova EE, Glinushkin AP. Species diversity in the Fusarium solani (Neocosmospora) complex and their pathogenicity for plants and humans. Doklady Biol Sci. 2022;507(1):416–27. 10.1134/S0012496622060217 36781537

[52] Yang X, Cheng J, Franks AE, Huang X, Yang Q, Cheng Z, et al. Loss of microbial diversity weakens specific soil functions, but increases soil ecosystem stability. Soil Biol Biochem. 2023;177:108916.

[53] Fanin N, Gundale MJ, Farrell M, Ciobanu M, Baldock JA, Nilsson M-C, et al. Consistent effects of biodiversity loss on multifunctionality across contrasting ecosystems. Nat Ecol Evol. 2018;2(2):269–78. 10.1038/s41559-017-0415-0 29255299

[54] Nawaz M, Sun J, Shabbir S, Khattak WA, Ren G, Nie X, et al. A review of plants strategies to resist biotic and abiotic environmental stressors. Sci Total Environ. 2023;900:165832. 10.1016/j.scitotenv.2023.165832 37524179

[55] Zhang H, Zhu J, Gong Z, Zhu J-K. Abiotic stress responses in plants. Nat Rev Genet. 2022;23(2):104–19. 10.1038/s41576-021-00413-0 34561623

[56] Bäumler AJ, Sperandio V. Interactions between the microbiota and pathogenic bacteria in the gut. Nature. 2016;535(7610):85–93. 10.1038/nature18849 PMC5114849 27383983

[57] Crowl TA, Crist TO, Parmenter RR, Belovsky G, Lugo AE. The spread of invasive species and infectious disease as drivers of ecosystem change. Front Ecol Environ. 2008;6(5):238–46.

[58] Das PP, Singh KRB, Nagpure G, Mansoori A, Singh RP, Ghazi IA, et al. Plant–soil–microbes: a tripartite interaction for nutrient acquisition and better plant growth for sustainable agricultural practices. Environ Res. 2022;214:113821. 10.1016/j.envres.2022.113821 35810815

[59] Trivedi P, Batista BD, Bazany KE, Singh BK. Plant–microbiome interactions under a changing world: responses, consequences and perspectives. N Phytol. 2022;234(6):1951–9. 10.1111/nph.18016 35118660

[60] Ahmad M, Sharma P, Rathee S, Singh HP, Batish DR, Lone GR, et al. Niche width analyses facilitate identification of high-risk endemic species at high altitudes in western Himalayas. Ecol Indic. 2021;126:107653.

[61] Babajanyan SG, Garushyants SK, Wolf YI, Koonin EV. Microbial diversity and ecological complexity emerging from environmental variation and horizontal gene transfer in a simple mathematical model. BMC Biol. 2024;22(1):148. 10.1186/s12915-024-01937-7 PMC11225191 38965531

[62] Lin Q, Wang Y, Li M, Xu Z, Li L. Ecological niche selection shapes the assembly and diversity of microbial communities in Casuarina equisetifolia L. Front Plant Sci. 2022;13:988485. 10.3389/fpls.2022.988485 PMC9632346 36340378

[63] de Celis M, Duque J, Marquina D, Salvadó H, Serrano S, Arregui L, et al. Niche differentiation drives microbial community assembly and succession in full-scale activated sludge bioreactors. NPJ Biofilms Microbiomes. 2022;8(1):23. 10.1038/s41522-022-00291-2 PMC9001656 35411053

[64] Sánchez González I, Hopper GW, Bucholz JR, Kubala ME, Lozier JD, Atkinson CL. Niche specialization and community niche space increase with species richness in filter-feeder assemblages. Ecosphere. 2023;14(5):e4495.

[65] Dangl JL, Jones JDG. Plant pathogens and integrated defence responses to infection. Nature. 2001;411(6839):826–33. 10.1038/35081161 11459065

[66] Shu W-S, Huang L-N. Microbial diversity in extreme environments. Nat Rev Microbiol. 2022;20(4):219–35. 10.1038/s41579-021-00648-y 34754082

[67] Wang Y, Pruitt RN, Nürnberger T, Wang Y. Evasion of plant immunity by microbial pathogens. Nat Rev Microbiol. 2022;20(8):449–64. 10.1038/s41579-022-00710-3 35296800

[68] Ahmad NB, Jaafaru MS, Isa Z, Abdulhamid Y, Kakudi RA, Ugya AY, et al. High pollution loads engineer oxygen dynamics, ecological niches, and pathogenicity shifts in freshwater environments. J Hazard Mater Adv. 2024;14:100425.

[69] McCullers JA. The co-pathogenesis of influenza viruses with bacteria in the lung. Nat Rev Microbiol. 2014;12(4):252–62. 10.1038/nrmicro3231 24590244

[70] Venter F, Matthews KR, Silvester E. Parasite co-infection: an ecological, molecular and experimental perspective. Proc R Soc B: Biol Sci. 2022;289(1967):20212155. 10.1098/rspb.2021.2155 PMC8767208 35042410

[71] Stegen JC, Lin X, Konopka AE, Fredrickson JK. Stochastic and deterministic assembly processes in subsurface microbial communities. ISME J. 2012;6(9):1653–64. 10.1038/ismej.2012.22 PMC3498916 22456445

[72] Matthews TJ, Whittaker RJ. Neutral theory and the species abundance distribution: recent developments and prospects for unifying niche and neutral perspectives. Ecol Evol. 2014;4(11):2263–77. 10.1002/ece3.1092 PMC4201439 25360266

[73] Limoli DH, Jones CJ, Wozniak DJ. Bacterial extracellular polysaccharides in biofilm formation and function. Microbiol Spectr. 2015;3:1128. 10.1128/microbiolspec.MB-0011-2014 PMC4657554 26185074

[74] Halperin T. Niches and ecological neutrality. Synthese. 2023;202(3):69.

[75] Levine JM, Bascompte J, Adler PB, Allesina S. Beyond pairwise mechanisms of species coexistence in complex communities. Nature. 2017;546(7656):56–64. 10.1038/nature22898 28569813

[76] Turnbull LA, Levine JM, Loreau M, Hector A. Coexistence, niches and biodiversity effects on ecosystem functioning. Ecol Lett. 2013;16(s1):116–27. 10.1111/ele.12056 23279851

[77] Hibbing ME, Fuqua C, Parsek MR, Peterson SB. Bacterial competition: Surviving and thriving in the microbial jungle. Nat Rev Microbiol. 2010;8(1):15–25. 10.1038/nrmicro2259 PMC2879262 19946288

[78] Buche L, Spaak JW, Jarillo J, De Laender F. Niche differences, not fitness differences, explain predicted coexistence across ecological groups. J Ecol. 2022;110(11):2785–96.

[79] Muthukrishnan R, Hansel-Welch N, Larkin DJ. Environmental filtering and competitive exclusion drive biodiversity-invasibility relationships in shallow lake plant communities. J Ecol. 2018;106(5):2058–70.

[80] Powell JR, Karunaratne S, Campbell CD, Yao H, Robinson L, Singh BK. Deterministic processes vary during community assembly for ecologically dissimilar taxa. Nat Commun. 2015;6(1):8444. 10.1038/ncomms9444 PMC4600744 26436640

[81] Dini-Andreote F, Stegen JC, van Elsas JD, Salles JF. Disentangling mechanisms that mediate the balance between stochastic and deterministic processes in microbial succession. Proc Natl Acad Sci. 2015;112(11):E1326–32. 10.1073/pnas.1414261112 PMC4371938 25733885

[82] Gao M, Xiong C, Gao C, Tsui CKM, Wang M-M, Zhou X, et al. Disease-induced changes in plant microbiome assembly and functional adaptation. Microbiome. 2021;9(1):187. 10.1186/s40168-021-01138-2 PMC8444440 34526096

[83] Pereira LB, Thomazella DPT, Teixeira PJPL. Plant–microbiome crosstalk and disease development. Curr Opin Plant Biol. 2023;72:102351. 10.1016/j.pbi.2023.102351 36848753

[84] Howard MM, Bass E, Chautá A, Mutyambai D, Kessler A. Integrating plant-to-plant communication and rhizosphere microbial dynamics: ecological and evolutionary implications and a call for experimental rigor. ISME J. 2022;16(1):5–9. 10.1038/s41396-021-01063-0 PMC8692333 34333553

[85] Solomon W, Janda T, Molnár Z. Unveiling the significance of rhizosphere: implications for plant growth, stress response, and sustainable agriculture. Plant Physiol Biochem. 2024;206:108290. 10.1016/j.plaphy.2023.108290 38150841

[86] Sethi A, Scharf ME. Biofuels: fungal, bacterial and insect degraders of lignocellulose. eLS. 2018;57–71.

[87] King BC, Waxman KD, Nenni NV, Walker LP, Bergstrom GC, Gibson DM. Arsenal of plant cell wall degrading enzymes reflects host preference among plant pathogenic fungi. Biotechnol Biofuels. 2011;4(1):4. 10.1186/1754-6834-4-4 PMC3051899 21324176

[88] König A, Müller R, Mogavero S, Hube B. Fungal factors involved in host immune evasion, modulation and exploitation during infection. Cell Microbiol. 2021;23(1):e13272. 10.1111/cmi.13272 32978997

[89] Waqar S, Bhat AA, Khan AA. Endophytic fungi: unravelling plant-endophyte interaction and the multifaceted role of fungal endophytes in stress amelioration. Plant Physiol Biochem. 2024;206:108174. 10.1016/j.plaphy.2023.108174 38070242

[90] Hernandez DJ, David AS, Menges ES, Searcy CA, Afkhami ME. Environmental stress destabilizes microbial networks. ISME J. 2021;15(6):1722–34. 10.1038/s41396-020-00882-x PMC8163744 33452480

[91] Deutschmann IM, Lima-Mendez G, Krabberød AK, Raes J, Vallina SM, Faust K, et al. Disentangling environmental effects in microbial association networks. Microbiome. 2021;9(1):232. 10.1186/s40168-021-01141-7 PMC8620190 34823593

[92] Rather MA, Gupta K, Mandal M. Microbial biofilm: formation, architecture, antibiotic resistance, and control strategies. Braz J Microbiol. 2021;52(4):1701–18. 10.1007/s42770-021-00624-x PMC8578483 34558029

[93] Lovering AL, Sockett RE. Microbe profile: Bdellovibrio bacteriovorus: a specialized bacterial predator of bacteria. Microbiology. 2021;167:001043. 10.1099/mic.0.001043 PMC8289219 33843574

[94] Du Toit A. Bdellovibrio bacteriovorus finds its prey. Nat Rev Microbiol. 2024;22(3):119. 10.1038/s41579-024-01012-6 38243075

[95] Lu Z, Kvammen A, Li H, Hao M, Inman AR, Bulone V, et al. A polysaccharide utilization locus from Chitinophaga pinensis simultaneously targets chitin and β-glucans found in fungal cell walls. mSphere. 2023;8:00028-32. 10.1128/msphere.00244-23 PMC10449523 37493618

