# Supplementary material for: Fungal and bacterial pathogenic co-infections mainly lead to the assembly of microbial community in tobacco stems
Source: Open Life Sci. 2025 Sep 20;20(1):20251103. doi: 10.1515/biol-2025-1103 (PMC12451430; doi:10.1515/biol-2025-1103)
Supplement: Supplementary material [file biol-2025-1103-sm.pdf]

Supplementary material

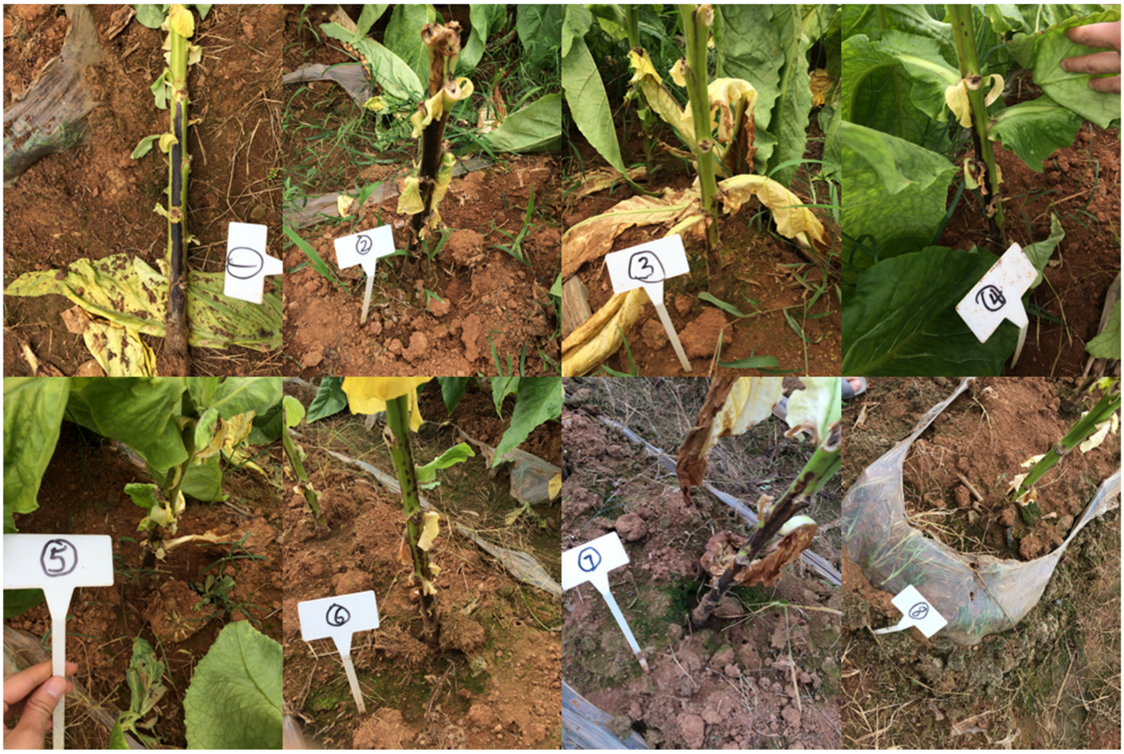

Figure S1: Symptoms of co-infection samples.

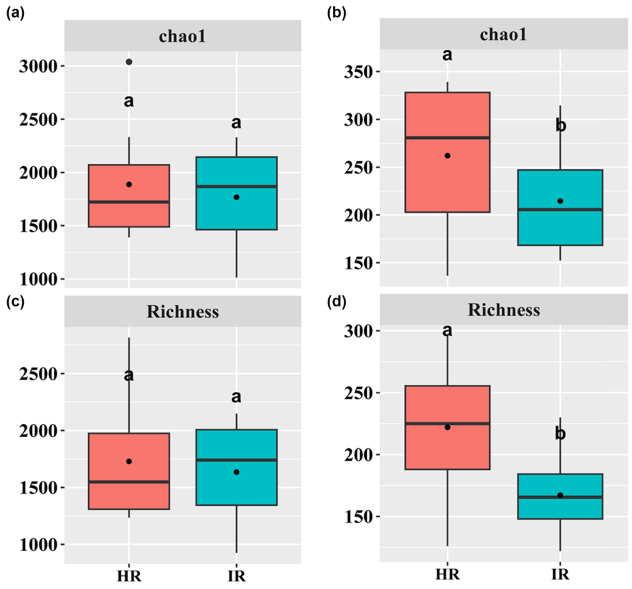

Figure S2: Alpha diversity indices of bacterial and fungal communities. (a) and (b) display the Chao1 index and species richness for bacterial communities in healthy stems (HR) and infected stems (IR), respectively. The same letter indicates no significant differences between HR and IR. (c) and (d) show the Chao1 index and species richness for fungal communities.

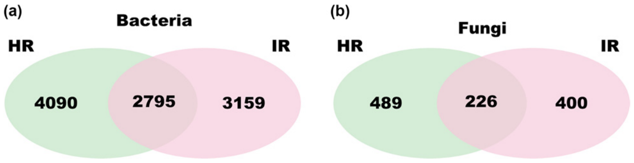

Figure S3: Venn plot of bacterial and fungal community of health and infected stems.

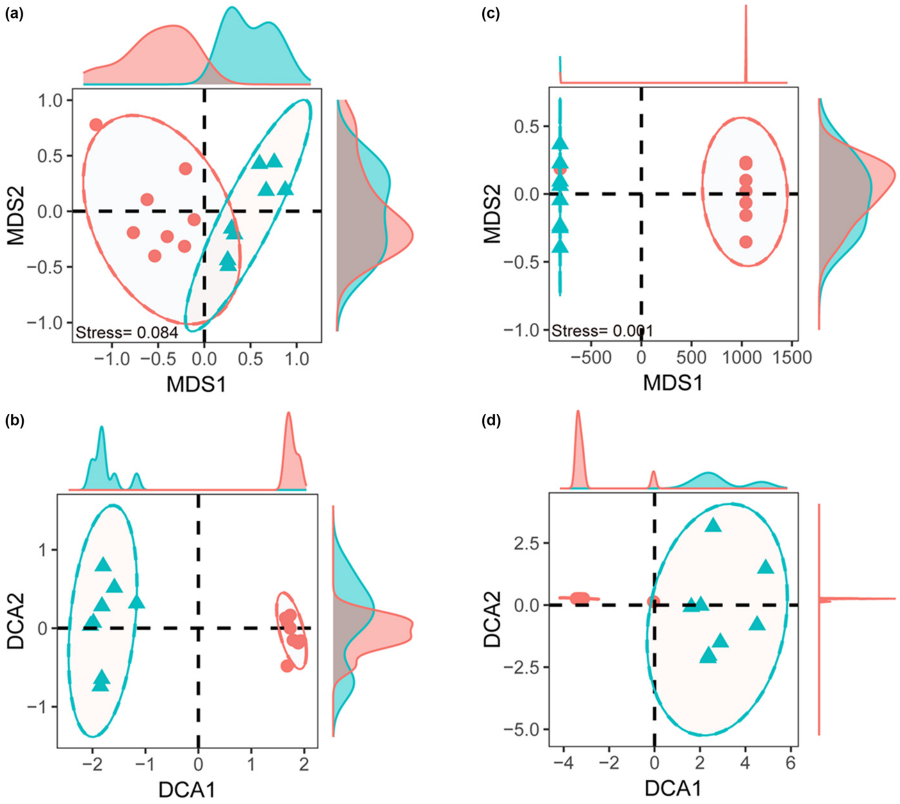

Figure S4: The beta diversity of bacterial and fungal community. Visualized through non-metric multidimensional scaling (NMDS) based on Bray–Curtis dissimilarity and detrended correspondence analysis (DCA) plots. The blue triangles represent HR while red circles represent IR.

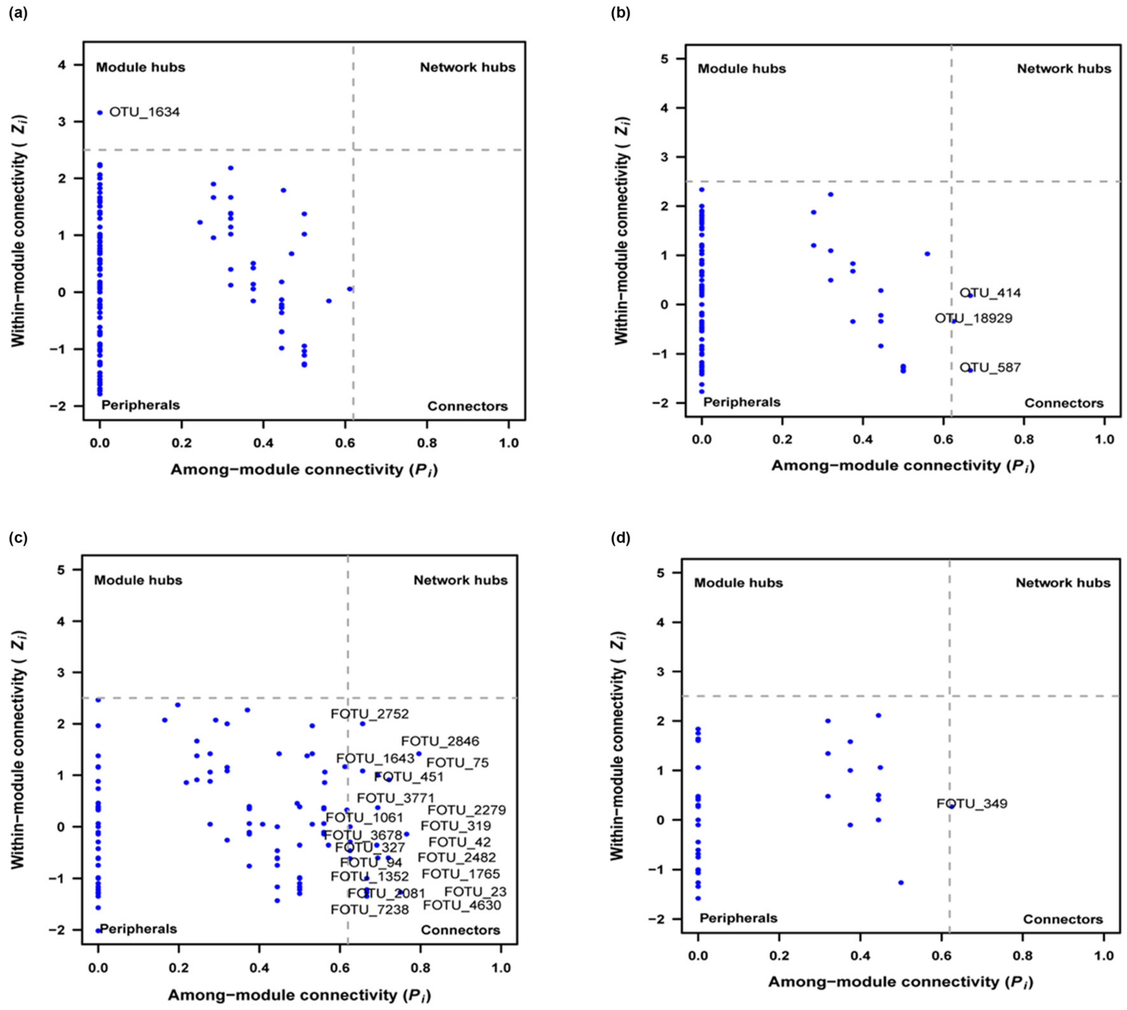

Figure S5: Z_i-P_i plots of network connectivity for bacterial and fungal communities. This figure presents Z_i-P_i plots from network analysis, displaying the within-module connectivity (Z_i) versus among-module connectivity (P_i) for bacterial and fungal operational taxonomic units (OTUs) in healthy and infected stems. (a) Bacterial community in healthy stems (HR). (b) Bacterial community in infected stems (IR). (c) Fungal community in healthy stems (HR). (d) Fungal community in infected stems (IR).

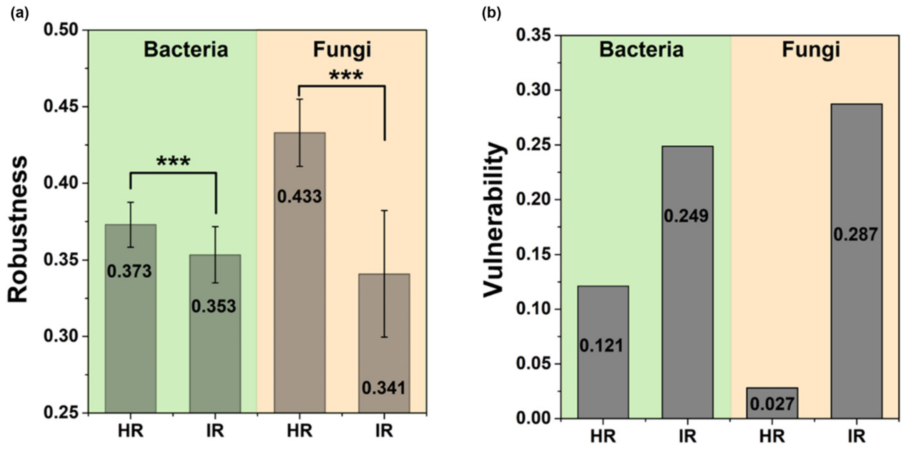

Figure S6: Robustness and vulnerability analysis for bacterial and fungal communities between health and infected stems. Robustness measured as the proportion of taxa remaining after 50% of the taxa are randomly removed from each of the empirical MENs. (a) The robustness of bacterial and fungal communities. (b) The vulnerability of bacterial and fungal communities. All model and images were calculated and generated by RStudio software. "***" mean the significantly differed at the level of $P < 0.001$.

Table S1: The dissimilarity analysis between health and infected endospheric community of bacteria

		MRPP		ANOSIM		PERMANOVA	
		**Delta**	***p***	***r***	***p***	***F***	***p***
HR VS. IR	Bray-curtis	0.3835	0.001***	1	0.002**	36.4602	0.001***
	Jaccard	0.7745	0.002**	0.6411	0.001***	2.3851	0.001***

Table S2: The dissimilarity analysis between health and infected endospheric community of fungi

		MRPP		ANOSIM		PERMANOVA	
		**Delta**	***p***	***r***	***p***	***F***	***p***
HR VS. IR	Bray-curtis	0.6217	0.001***	0.8942	0.001***	7.7881	0.001***
	Jaccard	0.7246	0.002**	0.9525	0.001***	5.6193	0.001***

Table S3: The network properties of bacterial community network

		HR	IR
	Cutoff	0.91	0.91
	Total nodes	358	345
	Total links	449	368
Emperical network	*R* square of power-law	0.794879623	0.877726936
	Average degree (avgK)	2.508379888	2.133333333
	Average clustering coefficient (avgCC)	0.208014099	0.195817805
	Average path distance (GD)	9.014771922	11.21989676
	Transitivity (Trans)	0.284386617	0.329956585
	Connectedness (Con)	0.529677793	0.179558477
	Modularity (fast_greedy)	0.872	0.924
Random network	Average clustering coefficient (avgCC)	0.001 ± 0.002	0.001 ± 0.002
	Average path distance (GD)	6.473 ± 0.127	8.037 ± 0.414
	Transitivity (Trans)	0.008 ± 0.005	0.006 ± 0.005
	Connectedness (Con)	0.871 ± 0.036	0.717 ± 0.047
	Modularity (fast_greedy)	0.719 ± 0.007	0.806 ± 0.009

Table S4: The network properties of fungal community network

		HR	IR
	Cutoff	0.85	0.85
	Total nodes	157	83
	Total links	348	93
Emperical network	*R* square of power-law	0.782467567	0.987997733
	Average degree (avgK)	4.433121019	2.240963855
	Average clustering coefficient (avgCC)	0.193547359	0.120596672
	Average path distance (GD)	3.898497469	7.595076401
	Transitivity (Trans)	0.193929174	0.184834123
	Connectedness (Con)	1	0.692330297
	Modularity (fast_greedy)	0.588	0.75
Random network	Average clustering coefficient (avgCC)	0.01 ± 0.007	0.003 ± 0.006
	Average path distance (GD)	3.507 ± 0.036	4.897 ± 0.37
	Transitivity (Trans)	0.04 ± 0.008	0.032 ± 0.021
	Connectedness (Con)	0.991 ± 0.014	0.758 ± 0.092
	Modularity (fast_greedy)	0.447 ± 0.01	0.681 ± 0.016

Table S5: The network properties of bipartite communities' networks

	HR	IR
Connectance	0.04295853	0.038719342
Links per species	4.79379562	2.972709552
Cluster coefficient	0.035714286	0.038186158
Nestedness	5.338376063	6.326398656
Weighted nestedness	0.287919002	0.352677213
Linkage density	21.25123715	14.28
Weighted connectance	0.03877963	0.027836257
Number of fungi	156	94
Number of bacteria	392	419
Mean number of shared partners of fungi	1.589991729	0.7558911
Mean number of shared partners of bacteria	0.443355603	0.193534389
Cluster coefficient of fungi	0.04295853	0.038719342
Cluster coefficient of bacteria	0.04295853	0.038719342
C score of fungi	0.856718201	0.910797196
C score of bacteria	0.894070724	0.90974964
V ratio of fungi	11.14475822	7.424724702
V ratio of bacteria	9.590761288	1.792883487
Robustness of fungi	0.772665837	0.726969438
Robustness of bacteria	0.880713104	0.876738295
Functional complementarity of fungi	611.0682894	401.3820386
Functional complementarity of bacteria	826.9709818	612.4956511
Vulnerability of bacteria	6.701530612	3.639618138
Modularity	0.426023	0.5453128

Table S6: The role and classification of keystone species in bipartite community network

Network_roles	OTUID	Domain	Phylum	Class	Order	Family	Genus	Species
Connector hubs	OTU_242	Bacteria	Proteobacteria	Gammaproteobacteria	Burkholderiales	Comamonadaceae	Acidovorax	Acidovorax sp. 1608163
Connector hubs	OTU_1351	Bacteria	Proteobacteria	Gammaproteobacteria	Pseudomonadales	Moraxellaceae	Acinetobacter	Acinetobacter johnsonii
Connector hubs	FOTU_1386	Fungi	Ascomycota	Sordariomycetes	Hypocreales	Nectriaceae	Acremoniopsis	Acremoniopsis suttonii
Connector hubs	OTU_1727	Bacteria	Actinobacteriota	Actinobacteria	Micrococcales	Microbacteriaceae	Agromyces	Agromyces mediolanus
Connector hubs	OTU_19537	Bacteria	Actinobacteriota	Actinobacteria	Micrococcales	Micrococcaceae	Arthrobacter	Arthrobacter globiformis
Connector hubs	OTU_16415	Bacteria	Firmicutes	Bacilli	Bacillales	Planococcaceae	Unclassified	Bacillus sp. FJAT-22090
Connector hubs	OTU_495	Bacteria	Proteobacteria	Alphaproteobacteria	Caulobacterales	Caulobacteraceae	Caulobacter	Caulobacter vibrioides
Connector hubs	OTU_102	Bacteria	Proteobacteria	Alphaproteobacteria	Rhizobiales	Devosiaceae	Devosia	Devosia riboflavina
Connector hubs	OTU_127	Bacteria	Proteobacteria	Alphaproteobacteria	Rhizobiales	Rhizobiaceae	Ensifer	Ensifer adhaerens
Connector hubs	OTU_1302	Bacteria	Proteobacteria	Alphaproteobacteria	Rhizobiales	Rhizobiaceae	Ensifer	Ensifer adhaerens
Module hubs	FOTU_2279	Fungi	Basidiomycota	Agaricomycetes	Agaricales	Entolomataceae	Entoloma	Entoloma mirum
Connector hubs	OTU_1110	Bacteria	Proteobacteria	Gammaproteobacteria	Enterobacterales	Enterobacteriaceae	Escherichia	Escherichia albertii
Connector hubs	OTU_2125	Bacteria	Proteobacteria	Gammaproteobacteria	Enterobacterales	Enterobacteriaceae	Escherichia	Escherichia coli O157:H7
Module hubs	FOTU_1677	Fungi	Basidiomycota	Agaricomycetes	Geastrales	Geastraceae	Geastrum	Geastrum brunneocapillatum
Connector hubs	OTU_128	Bacteria	Actinobacteriota	Actinobacteria	Pseudonocardiales	Pseudonocardiaceae	Kibdelosporangium	Kibdelosporangium phytohabitans
Connector hubs	OTU_1262	Bacteria	Proteobacteria	Gammaproteobacteria	Enterobacterales	Enterobacteriaceae	Enterobacter	Leclercia adecarboxylata
Connector hubs	OTU_1349	Bacteria	Proteobacteria	Gammaproteobacteria	Xanthomonadales	Xanthomonadaceae	Lysobacter	Lysobacter enzymogenes
Connector hubs	OTU_11553	Bacteria	Proteobacteria	Gammaproteobacteria	Burkholderiales	Oxalobacteraceae	Massilia	Massilia sp. YMA4
Connector hubs	OTU_183	Bacteria	Proteobacteria	Gammaproteobacteria	Burkholderiales	Oxalobacteraceae	Massilia	Massilia sp. YMA4
Connector hubs	FOTU_49	Fungi	Ascomycota	Sordariomycetes	Hypocreales	Clavicipitaceae	Metacordyceps	Metacordyceps chlamydosporia
Module hubs	FOTU_211	Fungi	Ascomycota	Sordariomycetes	Myrmecridiales	Myrmecridiaceae	Myrmecridium	Myrmecridium schulzeri
Module hubs	FOTU_647	Fungi	Ascomycota	Sordariomycetes	Hypocreales	Bionectriaceae	Nectriopsis	Nectriopsis lindauiana
Connector hubs	OTU_3050	Bacteria	Actinobacteriota	Actinobacteria	Corynebacteriales	Nocardiaceae	Nocardia	Nocardia cyriacigeorgica
Connector hubs	OTU_2504	Bacteria	Proteobacteria	Gammaproteobacteria	Burkholderiales	Oxalobacteraceae	Oxalicibacterium	Oxalicibacterium solurbis
Connector hubs	FOTU_132	Fungi	Ascomycota	Sordariomycetes	Phomatosporales	Phomatosporaceae	Phomatospora	Phomatospora biseriata
Connector hubs	OTU_63	Bacteria	Proteobacteria	Gammaproteobacteria	Xanthomonadales	Xanthomonadaceae	Pseudoxanthomonas	Pseudoxanthomonas mexicana
Connector hubs	FOTU_4888	Fungi	Kickxellomycota	Kickxellomycetes	Kickxellales	Kickxellaceae	Ramicandelaber	Ramicandelaber longisporus
Connector hubs	OTU_25612	Bacteria	Proteobacteria	Gammaproteobacteria	Burkholderiales	Comamonadaceae	Roseateles	Roseateles depolymerans

(*Continued*)

Table S6: *Continued*

Network_roles	OTUID	Domain	Phylum	Class	Order	Family	Genus	Species
Connector hubs	OTU_8	Bacteria	Proteobacteria	Alphaproteobacteria	Sphingomonadales	Sphingomonadaceae	Sphingomonas	Sphingomonas sp.
Connector hubs	FOTU_1073	Fungi	Ascomycota	Sordariomycetes	Hypocreales	Nectriaceae	Unclassified	Unclassified
Connector hubs	FOTU_1681	Fungi	Ascomycota	Unclassified	Unclassified	Unclassified	Unclassified	Unclassified
Connector hubs	FOTU_2081	Fungi	Unclassified	Unclassified	Unclassified	Unclassified	Unclassified	Unclassified
Connector hubs	FOTU_2347	Fungi	Chytridiomycota	Unclassified	Unclassified	Unclassified	Unclassified	Unclassified
Connector hubs	FOTU_275	Fungi	Mortierellomycota	Mortierellomycetes	Mortierellales	Unclassified	Unclassified	Unclassified
Connector hubs	FOTU_3000	Fungi	Chytridiomycota	Unclassified	Unclassified	Unclassified	Unclassified	Unclassified
Connector hubs	FOTU_3010	Fungi	Ascomycota	Eurotiomycetes	Eurotiales	Trichocomaceae	Talaromyces	Unclassified
Connector hubs	FOTU_313	Fungi	Ascomycota	Leotiomycetes	Helotiales	Helotiaceae	Scytalidium	Unclassified
Connector hubs	FOTU_319	Fungi	Unclassified	Unclassified	Unclassified	Unclassified	Unclassified	Unclassified
Connector hubs	FOTU_3216	Fungi	Ascomycota	Pezizomycetes	Pezizales	Ascobolaceae	Ascobolus	Unclassified
Connector hubs	FOTU_54	Fungi	Unclassified	Unclassified	Unclassified	Unclassified	Unclassified	Unclassified
Connector hubs	FOTU_55	Fungi	Ascomycota	Laboulbeniomycetes	Pyxidiophorales	Unclassified	Unclassified	Unclassified
Connector hubs	FOTU_98	Fungi	Ascomycota	Sordariomycetes	Sordariales	Chaetomiaceae	Unclassified	Unclassified
Connector hubs	OTU_1	Bacteria	Proteobacteria	Alphaproteobacteria	Rickettsiales	Mitochondria	Unclassified	Unclassified
Connector hubs	OTU_1058	Bacteria	Patescibacteria	Saccharimonadia	Saccharimonadales	Saccharimonadaceae	TM7a	Unclassified
Connector hubs	OTU_107	Bacteria	Proteobacteria	Gammaproteobacteria	Enterobacterales	Unclassified	Unclassified	Unclassified
Connector hubs	OTU_1150	Bacteria	Actinobacteriota	Actinobacteria	Micrococcales	Micrococcaceae	Pseudarthrobacter	Unclassified
Connector hubs	OTU_12281	Bacteria	Firmicutes	Bacilli	Bacillales	Planococcaceae	Domibacillus	Unclassified
Connector hubs	OTU_1440	Bacteria	Actinobacteriota	Thermoleophilia	Gaiellales	Gaiellaceae	Gaiella	Unclassified
Connector hubs	OTU_14599	Bacteria	Proteobacteria	Gammaproteobacteria	Pseudomonadales	Pseudomonadaceae	Pseudomonas	Unclassified
Connector hubs	OTU_158	Bacteria	Proteobacteria	Gammaproteobacteria	Steroidobacterales	Steroidobacteraceae	Steroidobacter	Unclassified
Connector hubs	OTU_1700	Bacteria	Actinobacteriota	Actinobacteria	Streptomycetales	Streptomycetaceae	Streptomyces	Unclassified
Connector hubs	OTU_19460	Bacteria	Proteobacteria	Alphaproteobacteria	Sphingomonadales	Sphingomonadaceae	Sphingopyxis	Unclassified
Connector hubs	OTU_199	Bacteria	Proteobacteria	Gammaproteobacteria	Steroidobacterales	Steroidobacteraceae	Steroidobacter	Unclassified
Connector hubs	OTU_2160	Bacteria	Bacteroidota	Bacteroidia	Chitinophagales	Chitinophagaceae	Chitinophaga	Unclassified
Connector hubs	OTU_22331	Bacteria	Proteobacteria	Gammaproteobacteria	Burkholderiales	Alcaligenaceae	Achromobacter spanius	Unclassified
Connector hubs	OTU_2372	Bacteria	Proteobacteria	Alphaproteobacteria	Rickettsiales	Mitochondria	Unclassified	Unclassified

(*Continued*)

Table S6: *Continued*

Network_roles	OTUID	Domain	Phylum	Class	Order	Family	Genus	Species
Connector hubs	OTU_24375	Bacteria	Proteobacteria	Alphaproteobacteria	Rickettsiales	Mitochondria	Nicotiana sylvestris	Unclassified
Connector hubs	OTU_25589	Bacteria	Proteobacteria	Gammaproteobacteria	Burkholderiales	Rhodocyclaceae	Sulfuritalea	Unclassified
Connector hubs	OTU_3257	Bacteria	Patescibacteria	Saccharimonadia	Saccharimonadales	Unclassified	Unclassified	Unclassified
Connector hubs	OTU_3506	Bacteria	Proteobacteria	Alphaproteobacteria	Rhizobiales	Devosiaceae	Devosia	Unclassified
Connector hubs	OTU_36737	Bacteria	Proteobacteria	Alphaproteobacteria	Rickettsiales	Mitochondria	Unclassified	Unclassified
Connector hubs	OTU_37689	Bacteria	Patescibacteria	Saccharimonadia	Saccharimonadales	Unclassified	Unclassified	Unclassified
Connector hubs	OTU_3914	Bacteria	Proteobacteria	Alphaproteobacteria	Sphingomonadales	Sphingomonadaceae	Sphingomonas	Unclassified
Connector hubs	OTU_405	Bacteria	Actinobacteriota	Actinobacteria	Micrococcales	Micrococcaceae	Pseudarthrobacter	Unclassified
Connector hubs	OTU_409	Bacteria	Proteobacteria	Gammaproteobacteria	Burkholderiales	Oxalobacteraceae	Unclassified	Unclassified
Connector hubs	OTU_5538	Bacteria	Actinobacteriota	Actinobacteria	Streptomycetales	Streptomycetaceae	Streptomyces	Unclassified
Connector hubs	OTU_591	Bacteria	Proteobacteria	Alphaproteobacteria	Sphingomonadales	Sphingomonadaceae	Novosphingobium	Unclassified
Connector hubs	OTU_6114	Bacteria	Unclassified	Unclassified	Unclassified	Unclassified	Unclassified	Unclassified
Connector hubs	OTU_669	Bacteria	Firmicutes	Bacilli	Bacillales	Bacillaceae	Bacillus	Unclassified
Connector hubs	OTU_699	Bacteria	Proteobacteria	Alphaproteobacteria	Rhizobiales	Devosiaceae	Devosia	Unclassified
Connector hubs	OTU_72	Bacteria	Proteobacteria	Alphaproteobacteria	Rickettsiales	Mitochondria	Unclassified	Unclassified
Connector hubs	OTU_8074	Bacteria	Actinobacteriota	Actinobacteria	Streptomycetales	Streptomycetaceae	Streptomyces	Unclassified
Connector hubs	OTU_8186	Bacteria	Actinobacteriota	Actinobacteria	Propionibacteriales	Nocardioidaceae	Nocardioides	Unclassified
Connector hubs	OTU_943	Bacteria	Actinobacteriota	Thermoleophilia	Solirubrobacterales	Unclassified	Unclassified	Unclassified
Module hubs	FOTU_154	Fungi	Ascomycota	Unclassified	Unclassified	Unclassified	Unclassified	Unclassified
Module hubs	FOTU_1590	Fungi	Unclassified	Unclassified	Unclassified	Unclassified	Unclassified	Unclassified
Module hubs	FOTU_1632	Fungi	Unclassified	Unclassified	Unclassified	Unclassified	Unclassified	Unclassified
Module hubs	FOTU_164	Fungi	Ascomycota	Sordariomycetes	Sordariales	Chaetomiaceae	Unclassified	Unclassified
Module hubs	FOTU_1746	Fungi	Unclassified	Unclassified	Unclassified	Unclassified	Unclassified	Unclassified
Module hubs	FOTU_2508	Fungi	Unclassified	Unclassified	Unclassified	Unclassified	Unclassified	Unclassified
Module hubs	FOTU_69	Fungi	Ascomycota	Sordariomycetes	Unclassified	Unclassified	Unclassified	Unclassified
Module hubs	FOTU_779	Fungi	Chytridiomycota	Spizellomycetes	Spizellomycetales	Unclassified	Unclassified	Unclassified
Network hubs	FOTU_1094	Fungi	Chytridiomycota	Unclassified	Unclassified	Unclassified	Unclassified	Unclassified
Network hubs	FOTU_1841	Fungi	Unclassified	Unclassified	Unclassified	Unclassified	Unclassified	Unclassified

(*Continued*)

Table S6: *Continued*

Network_roles	OTUID	Domain	Phylum	Class	Order	Family	Genus	Species
Network hubs	FOTU_185	Fungi	Ascomycota	Sordariomycetes	Branch06	Unclassified	Unclassified	Unclassified
Network hubs	FOTU_2482	Fungi	Mucoromycota	Endogonomycetes	Endogonales	Unclassified	Unclassified	Unclassified
Network hubs	FOTU_29	Fungi	Ascomycota	Sordariomycetes	Microascales	Microascales	Vermiculariopsiella	Vermiculariopsiella hongkongensis
Connector hubs	FOTU_134	Fungi	Basidiomycota	Agaricomycetes	Agaricales	Stephanosporaceae	Unclassified	Unclassified
Connector hubs	FOTU_1940	Fungi	Basidiomycota	Agaricomycetes	Agaricales	Entolomataceae	Entoloma	Entoloma quellarense
Connector hubs	FOTU_274	Fungi	Chytridiomycota	Rhizophlyctidomycetes	Rhizophlyctidales	Rhizophlyctidaceae	Unclassified	Unclassified
Connector hubs	FOTU_456	Fungi	Unclassified	Unclassified	Unclassified	Unclassified	Unclassified	Unclassified
Connector hubs	FOTU_49	Fungi	Ascomycota	Sordariomycetes	Hypocreales	Clavicipitaceae	Metacordyceps	Metacordyceps chlamydosporia
Connector hubs	FOTU_58	Fungi	Mortierellomycota	Mortierellomycetes	Mortierellales	Mortierellaceae	Mortierella	Mortierella amoeboidea
Connector hubs	FOTU_592	Fungi	Unclassified	Unclassified	Unclassified	Unclassified	Unclassified	Unclassified
Connector hubs	FOTU_681	Fungi	Ascomycota	Orbiliomycetes	Orbiliales	Orbiliaceae	Arthrobotrys	Arthrobotrys arthrobotryoides
Connector hubs	FOTU_938	Fungi	Unclassified	Unclassified	Unclassified	Unclassified	Unclassified	Unclassified
Connector hubs	OTU_102	Bacteria	Proteobacteria	Alphaproteobacteria	Rhizobiales	Devosiaceae	Devosia	Devosia riboflavina
Connector hubs	OTU_1052	Bacteria	Proteobacteria	Alphaproteobacteria	Rhizobiales	Rhizobiaceae	Rhizobium	Rhizobium etli
Connector hubs	OTU_10731	Bacteria	Bdellovibrionota	Bdellovibrionia	Bdellovibrionales	Bdellovibrionaceae	Bdellovibrio	Bdellovibrio sp. oral clone CA006
Connector hubs	OTU_1106	Bacteria	Bacteroidota	Bacteroidia	Chitinophagales	Chitinophagaceae	Flavitalea	Unclassified
Connector hubs	OTU_11400	Bacteria	Bacteroidota	Bacteroidia	Unclassified	Unclassified	Unclassified	Unclassified
Connector hubs	OTU_1176	Bacteria	Proteobacteria	Alphaproteobacteria	Rhizobiales	Xanthobacteraceae	Tardiphaga	Unclassified
Connector hubs	OTU_1255	Bacteria	Bacteroidota	Bacteroidia	Chitinophagales	Chitinophagaceae	Unclassified	Unclassified
Connector hubs	OTU_1270	Bacteria	Proteobacteria	Gammaproteobacteria	Xanthomonadales	Xanthomonadaceae	Thermomonas	Unclassified
Connector hubs	OTU_1279	Bacteria	Proteobacteria	Alphaproteobacteria	Rhizobiales	Rhizobiaceae	Rhizobium	Rhizobium jaguaris
Connector hubs	OTU_1341	Bacteria	Bacteroidota	Bacteroidia	Cytophagales	Spirosomaceae	Dyadobacter	Dyadobacter fermentans
Connector hubs	OTU_13896	Bacteria	Proteobacteria	Gammaproteobacteria	Burkholderiales	Burkholderiaceae	Limnobacter	Unclassified
Connector hubs	OTU_1397	Bacteria	Proteobacteria	Alphaproteobacteria	Rhizobiales	Rhizobiaceae	Shinella	Unclassified
Connector hubs	OTU_1589	Bacteria	Bacteroidota	Bacteroidia	Chitinophagales	37-13	Unclassified	Unclassified
Connector hubs	OTU_167	Bacteria	Bacteroidota	Bacteroidia	Chitinophagales	Chitinophagaceae	Terrimonas	Terrimonas sp.
Connector hubs	OTU_17880	Bacteria	Proteobacteria	Gammaproteobacteria	Burkholderiales	Burkholderiaceae	Ralstonia	Ralstonia solanacearum
Connector hubs	OTU_1938	Bacteria	Firmicutes	Bacilli	Paenibacillales	Paenibacillaceae	Cohnella	Unclassified

(*Continued*)

Table S6: *Continued*

Network_roles	OTUID	Domain	Phylum	Class	Order	Family	Genus	Species
Connector hubs	OTU_2045	Bacteria	Proteobacteria	Alphaproteobacteria	Rhizobiales	Beijerinckiaceae	Bosea	Bosea thiooxidans
Connector hubs	OTU_2431	Bacteria	Proteobacteria	Gammaproteobacteria	Burkholderiales	Alcaligenaceae	Achromobacter	Unclassified
Connector hubs	OTU_24330	Bacteria	Proteobacteria	Alphaproteobacteria	Rhizobiales	Devosiaceae	Devosia	Unclassified
Connector hubs	OTU_25	Bacteria	Bacteroidota	Bacteroidia	Chitinophagales	Chitinophagaceae	Niastella	Unclassified
Connector hubs	OTU_26989	Bacteria	Proteobacteria	Gammaproteobacteria	Unclassified	Unclassified	Unclassified	Unclassified
Connector hubs	OTU_2839	Bacteria	Bacteroidota	Bacteroidia	Chitinophagales	Chitinophagaceae	Terrimonas	Unclassified
Connector hubs	OTU_2912	Bacteria	Bacteroidota	Bacteroidia	Sphingobacteriales	env.OPS 17	Unclassified	Unclassified
Connector hubs	OTU_305	Bacteria	Proteobacteria	Gammaproteobacteria	Xanthomonadales	Xanthomonadaceae	Stenotrophomonas	Stenotrophomonas acidaminiphila
Connector hubs	OTU_3077	Bacteria	Proteobacteria	Alphaproteobacteria	Sphingomonadales	Sphingomonadaceae	Sphingopyxis	Sphingopyxis macrogoltabida
Connector hubs	OTU_3175	Bacteria	Proteobacteria	Alphaproteobacteria	Caulobacterales	Caulobacteraceae	Asticcacaulis	Unclassified
Connector hubs	OTU_324	Bacteria	Proteobacteria	Alphaproteobacteria	Rhizobiales	Devosiaceae	Devosia	Unclassified
Connector hubs	OTU_3297	Bacteria	Bacteroidota	Bacteroidia	Sphingobacteriales	Sphingobacteriaceae	Pedobacter	Pedobacter solisilvae
Connector hubs	OTU_332	Bacteria	Proteobacteria	Gammaproteobacteria	Steroidobacterales	Steroidobacteraceae	Steroidobacter	metagenome
Connector hubs	OTU_3348	Bacteria	Bdellovibrionota	Bdellovibrionia	Bdellovibrionales	Bdellovibrionaceae	Bdellovibrio	Bdellovibrio bacteriovorus
Connector hubs	OTU_386	Bacteria	Proteobacteria	Alphaproteobacteria	Rhizobiales	Xanthobacteraceae	Rhodopseudomonas	Unclassified
Connector hubs	OTU_398	Bacteria	Bacteroidota	Bacteroidia	Chitinophagales	Chitinophagaceae	Pseudoflavitalea	Unclassified
Connector hubs	OTU_4245	Bacteria	Proteobacteria	Alphaproteobacteria	Rhizobiales	Xanthobacteraceae	Unclassified	Afipia genosp. 12
Connector hubs	OTU_4295	Bacteria	Bacteroidota	Bacteroidia	Chitinophagales	Chitinophagaceae	Terrimonas	Terrimonas sp.
Connector hubs	OTU_4639	Bacteria	Proteobacteria	Alphaproteobacteria	Rhizobiales	Rhizobiaceae	Aminobacter	Unclassified
Connector hubs	OTU_4741	Bacteria	Proteobacteria	Gammaproteobacteria	Burkholderiales	Burkholderiaceae	Unclassified	Unclassified
Connector hubs	OTU_493	Bacteria	Proteobacteria	Alphaproteobacteria	Caulobacterales	Caulobacteraceae	Caulobacter	Caulobacter sp.
Connector hubs	OTU_52	Bacteria	Proteobacteria	Gammaproteobacteria	Enterobacterales	Enterobacteriaceae	Cedecea	Cedecea neteri
Connector hubs	OTU_5380	Bacteria	Firmicutes	Bacilli	Paenibacillales	Paenibacillaceae	Cohnella	Unclassified
Connector hubs	OTU_55	Bacteria	Bacteroidota	Bacteroidia	Chitinophagales	Chitinophagaceae	Terrimonas	Unclassified
Connector hubs	OTU_550	Bacteria	Proteobacteria	Alphaproteobacteria	Rhizobiales	Rhizobiaceae	Shinella	Shinella granuli
Connector hubs	OTU_5646	Bacteria	Proteobacteria	Gammaproteobacteria	Burkholderiales	Burkholderiaceae	Unclassified	Unclassified
Connector hubs	OTU_5786	Bacteria	Bacteroidota	Bacteroidia	Sphingobacteriales	Sphingobacteriaceae	Sphingobacterium	Sphingobacterium composti Yoo et al. 2007 non Ten et al. 2007

(*Continued*)

Table S6: *Continued*

Network_roles	OTUID	Domain	Phylum	Class	Order	Family	Genus	Species
Connector hubs	OTU_6065	Bacteria	Proteobacteria	Gammaproteobacteria	Burkholderiales	Oxalobacteraceae	Oxalicibacterium	Oxalicibacterium solurbis
Connector hubs	OTU_63	Bacteria	Proteobacteria	Gammaproteobacteria	Xanthomonadales	Xanthomonadaceae	Pseudoxanthomonas	Pseudoxanthomonas mexicana
Connector hubs	OTU_645	Bacteria	Bacteroidota	Bacteroidia	Chitinophagales	Chitinophagaceae	Chitinophaga	Unclassified
Connector hubs	OTU_680	Bacteria	Proteobacteria	Alphaproteobacteria	Sphingomonadales	Sphingomonadaceae	Sphingopyxis	Unclassified
Connector hubs	OTU_720	Bacteria	Proteobacteria	Alphaproteobacteria	Sphingomonadales	Sphingomonadaceae	Sphingomonas	Unclassified
Connector hubs	OTU_728	Bacteria	Proteobacteria	Gammaproteobacteria	Burkholderiales	Methylophilaceae	Methylophilus	Methylophilus methylotrophus
Connector hubs	OTU_7627	Bacteria	Proteobacteria	Gammaproteobacteria	Burkholderiales	Burkholderiaceae	Unclassified	Unclassified
Connector hubs	OTU_8875	Bacteria	Proteobacteria	Gammaproteobacteria	Burkholderiales	Methylophilaceae	Unclassified	Unclassified
Connector hubs	OTU_91	Bacteria	Actinobacteriota	Actinobacteria	Micrococcales	Micrococcaceae	Pseudarthrobacter	Unclassified
Connector hubs	OTU_922	Bacteria	Bacteroidota	Bacteroidia	Chitinophagales	Chitinophagaceae	Chitinophaga	Chitinophaga arvensicola
Connector hubs	OTU_9481	Bacteria	Patescibacteria	Saccharimonadia	Saccharimonadales	Saccharimonadaceae	TM7a	Unclassified
Connector hubs	OTU_9545	Bacteria	Firmicutes	Bacilli	Paenibacillales	Paenibacillaceae	Paenibacillus	Paenibacillus sp. YN15
Connector hubs	OTU_97	Bacteria	Proteobacteria	Gammaproteobacteria	Burkholderiales	Comamonadaceae	Variovorax	Variovorax paradoxus
Module hubs	FOTU_1	Fungi	Basidiomycota	Agaricomycetes	Agaricales	Psathyrellaceae	Coprinellus	Coprinellus flocculosus
Module hubs	FOTU_117	Fungi	Chytridiomycota	Rhizophydiomycetes	Rhizophydiales	Terramycetaceae	Boothiomyces	Boothiomyces macroporosum
Module hubs	FOTU_121	Fungi	Unclassified	Unclassified	Unclassified	Unclassified	Unclassified	Unclassified
Module hubs	FOTU_142	Fungi	Rozellomycota	Unclassified	Unclassified	Unclassified	Unclassified	Unclassified
Module hubs	FOTU_1568	Fungi	Unclassified	Unclassified	Unclassified	Unclassified	Unclassified	Unclassified
Module hubs	FOTU_169	Fungi	Ascomycota	Eurotiomycetes	Onygenales	Onygenales	Chrysosporium	Chrysosporium lobatum
Module hubs	FOTU_25	Fungi	Ascomycota	Sordariomycetes	Hypocreales	Nectriaceae	Neocosmospora	Neocosmospora falciformis
Module hubs	FOTU_260	Fungi	Ascomycota	Orbiliomycetes	Orbiliales	Orbiliaceae	Orbilia	Unclassified
Module hubs	FOTU_288	Fungi	Unclassified	Unclassified	Unclassified	Unclassified	Unclassified	Unclassified
Module hubs	FOTU_3	Fungi	Ascomycota	Sordariomycetes	Hypocreales	Nectriaceae	Unclassified	Unclassified
Module hubs	FOTU_31	Fungi	Ascomycota	Dothideomycetes	Pleosporales	Leptosphaeriaceae	Unclassified	Unclassified
Module hubs	FOTU_317	Fungi	Ascomycota	Sordariomycetes	Hypocreales	Hypocreaceae	Hypomyces	Unclassified
Module hubs	FOTU_34	Fungi	Ascomycota	Sordariomycetes	Unclassified	Unclassified	Unclassified	Unclassified
Module hubs	FOTU_396	Fungi	Ascomycota	Orbiliomycetes	Unclassified	Unclassified	Unclassified	Unclassified
Module hubs	FOTU_397	Fungi	Ascomycota	Sordariomycetes	Sordariales	Lasiosphaeriaceae	Schizothecium	Unclassified

(*Continued*)

Table S6: *Continued*

Network_roles	OTUID	Domain	Phylum	Class	Order	Family	Genus	Species
Module hubs	FOTU_415	Fungi	Unclassified	Unclassified	Unclassified	Unclassified	Unclassified	Unclassified
Module hubs	FOTU_45	Fungi	Chytridiomycota	Unclassified	Unclassified	Unclassified	Unclassified	Unclassified
Module hubs	FOTU_479	Fungi	Unclassified	Unclassified	Unclassified	Unclassified	Unclassified	Unclassified
Module hubs	FOTU_55	Fungi	Ascomycota	Laboulbeniomycetes	Pyxidiophorales	Unclassified	Unclassified	Unclassified
Module hubs	FOTU_61	Fungi	Basidiomycota	Agaricomycetes	Agaricales	Psathyrellaceae	Psathyrella	Unclassified
Module hubs	FOTU_75	Fungi	Ascomycota	Sordariomycetes	Hypocreales	Hypocreales	Sarocladium	Sarocladium kiliense
Module hubs	FOTU_89	Fungi	Ascomycota	Pezizomycetes	Pezizales	Pyronemataceae	Lasiobolidium	Lasiobolidium orbiculoides
Network hubs	FOTU_171	Fungi	Chytridiomycota	Spizellomycetes	Spizellomycetales	Unclassified	Unclassified	Unclassified
Network hubs	FOTU_23	Fungi	Ascomycota	Dothideomycetes	Capnodiales	Cladosporiaceae	Cladosporium	Unclassified
